# Effects of nitrogen on floral scent and other reproductive traits in two closely related species of *Ipomopsis* and their hybrids

**DOI:** 10.1093/aob/mcag192

**Published:** 2026-07-02

**Authors:** Janelle A Bohey, Arturo A Aguilar, John M Powers, Diane R Campbell

**Affiliations:** Rocky Mountain Biological Laboratory, Crested Butte, CO 81224, USA; Department of Ecology and Evolutionary Biology, University of California, Irvine, CA 92697, USA; Rocky Mountain Biological Laboratory, Crested Butte, CO 81224, USA; Department of Organismic and Evolutionary Biology, Harvard University, Cambridge, MA 02138, USA; Rocky Mountain Biological Laboratory, Crested Butte, CO 81224, USA; Department of Ecology and Evolutionary Biology, University of California, Irvine, CA 92697, USA; Rocky Mountain Biological Laboratory, Crested Butte, CO 81224, USA; Department of Ecology and Evolutionary Biology, University of California, Irvine, CA 92697, USA

**Keywords:** Floral morphology, floral scent, *Ipomopsis aggregata* ssp. *aggregata*, *Ipomopsis tenuituba* ssp. *tenuituba*, nectar, phenotypic plasticity, volatile organic compound, nitrogen

## Abstract

**Background and Aims:**

Floral traits are key mediators of plant–pollinator interactions, and can be altered through changes in resources to plants. Although several studies have tested how nitrogen affects visual traits, only a few have examined nitrogen effects on floral scent, and to our knowledge none have evaluated the effect of nitrogen addition on nitrogenous volatile compounds. We test how nitrogen availability influences floral morphology, nectar traits, display size and floral scent (total emissions and individual compounds) during the day and night, with a special focus on a nitrogenous compound, indole, that influences reproductive isolation due to pollinator behaviour.

**Methods:**

We investigated the effects of supplementary nitrogen on floral traits in two closely related species of *Ipomopsis* and their hybrids in greenhouse and field environments in the Colorado Rocky Mountains. In the greenhouse, we studied *I. aggregata* and *I. tenuituba* collected from natural populations, while in the field we studied *I. aggregata* and hybrids. By combining greenhouse and field components, we assessed the robustness of floral trait responses to nitrogen availability.

**Key Results:**

Responses to nitrogen varied among taxa and between environments. In the field, nitrogen increased corolla width and nectar production in *I. aggregata* but not in hybrids. In the greenhouse, nitrogen decreased nectar production in *I. tenuituba*, but increased flower production and inflorescence height in *I. aggregata*. Emission composition showed a nitrogen effect in greenhouse *I. aggregata* but not in *I. tenuituba* or in either field taxon. Across both environments, total emissions and emission of indole were unaffected by nitrogen addition. Day–night differences explained most of the variation in total per-flower emissions, with compound-specific time differences contributing to this.

**Conclusions:**

Our results suggest that, even for close relatives, floral traits respond differently to nitrogen addition. They also suggest soil nitrogen has weaker effects on floral volatiles than on flower number and some other individual floral traits.

## INTRODUCTION

Nitrogen is widely recognized as a limiting nutrient in terrestrial ecosystems due to the constraints it places on plant growth and reproduction by affecting photosynthesis, protein synthesis and uptake of other key elements (Vitousek and Howarth, 1991; Zheng *et al.*, 2021). Extensive research demonstrates that nitrogen availability influences plant primary productivity (e.g. increased biomass), biochemistry (e.g. foliar N concentrations) and chemical defences (reviewed in Stevens *et al.*, 2018; Li *et al.*, 2024). Changes in soil nitrogen levels can also influence higher trophic level interactions through both primary and secondary metabolic processes (Gutschick, 1981; Lee and Rudge, 1986; Tilman, 1988; Li *et al.*, 2024). Indeed, several experiments link nutrient-driven secondary metabolic changes to altered insect behaviour and multitrophic interactions (reviewed in Li *et al.*, 2024). Some plant species prioritize defensive metabolite production, rather than growth, under low nitrogen. For example, in rice and eggplant (aubergine), nitrogen deficiencies increase levels of jasmonic acid, a principal modulator of plant defences against insect pests (Zheng *et al.*, 2021, 2024). While these effects of nitrogen levels on herbivore defence strategies are well documented, comparatively less attention has been given to how nitrogen affects floral traits that play a key role in mediating plant–pollinator interactions, such as nectar rewards, floral scent and floral morphology (Kevan *et al.*, 1993; Pellmyr, 2002; Raguso, 2008). Given the fundamental role of pollinators in maintaining biodiversity and ecosystem stability (Bascompte *et al.*, 2006), understanding how nitrogen may alter floral traits important for pollinator attraction is consequential for predicting how increases in nitrogen deposition as a result of anthropogenic climate change will impact species interactions.

Phenotypic plasticity in floral traits allows plants to respond to changes in their environments, but these plastic responses can also reshape how pollinators perceive and interact with flowers, ultimately influencing pollinator attraction and plant reproduction (Junker and Parachnowitsch, 2015; Yan *et al.*, 2016). Traits strongly associated with reproductive success, such as flower number and seed production, typically increase with nitrogen addition (Campbell and Halama, 1993; Asikainen and Mutikainen, 2005; Muñoz *et al.*, 2005; Burkle and Irwin, 2009, 2010; Friberg *et al.*, 2017; Spigler and Woodard, 2019; Gao *et al.*, 2023) with a few exceptions (Burkle and Irwin, 2010; Gijbels *et al.*, 2015; Brito Vera *et al.*, 2025). Flower production is one of the more well-documented trait responses to nitrogen addition, with other floral traits receiving far less attention. For example, only a handful of studies have examined the effects of nitrogen on nectar production. Low levels of nitrogen increased nectar production in *Antirrhinum majus* but had no effect in *Trifolium pratense*, *Linum lewisii* or *Potentilla pulcherrima* (Shuel, 1957; Burkle and Irwin, 2009, 2010). Compared with nectar, we know even less about how nitrogen availability influences floral scent, a key but understudied factor in plant–pollinator interactions (Raguso, 2008).

Floral scent is the result of volatile organic compounds (VOCs) released by flowers. To synthesize VOCs, plants use carbon, nitrogen, sulfur and energy from primary metabolism (Dudareva *et al.*, 2013). Therefore, in theory, when the availability of these resources shifts this could alter VOC production. While the biosynthetic pathways and regulatory mechanisms underlying floral VOC production are well characterized (Pichersky *et al.*, 2006; Dudareva *et al.*, 2013; Muhlemann *et al.*, 2014), this mechanistic understanding provides limited insight into how environmental factors, such as soil nutrient availability, influence VOC emission in natural settings. Only a handful of studies have looked at the effect of nutrients on floral volatiles, with mixed results (Friberg *et al.*, 2017; Majetic *et al.*, 2017; Luizzi *et al.*, 2021; Höfer *et al.*, 2022). For example, in *Lithophragma bolanderi*, nutrient level affected flower production and vegetative traits, but not floral scent composition or total emissions (Friberg *et al.*, 2017). In *Petunia hybrida*, addition of soil nitrogen increased emission rates for only one of the ten scent compounds analysed (Majetic *et al.*, 2017), whereas in *Arabis alpina* total emissions but not composition increased with nutrient (NPK) addition (Luizzi *et al.*, 2021). Across three Brassicaceae species, total floral scent emission rate was unaffected by nitrogen or watering treatments, but scent composition varied with nitrogen, with compound-specific responses that sometimes interacted with watering (Höfer *et al.*, 2022). As floral scent is important to pollinator attraction (Pichersky and Gershenzon, 2002; Knudsen *et al.*, 2004; Schiestl, 2010; Farré-Armengol *et al.*, 2015; Junker and Parachnowitsch, 2015; Kantsa *et al.*, 2018), the lack of information on how nitrogen availability affects floral volatile emissions makes it difficult to predict how changes in nutrient availability will influence plant–pollinator interactions via chemical signalling.

One unexplored aspect in the relationship between nitrogen and floral scent is how nitrogen availability specifically affects VOCs that contain nitrogen. Nitrogen addition could directly alter both the quantity and composition of floral scents. One potential avenue for this is by increasing the production of nitrogenous volatiles, or volatiles with nitrogen-containing precursors. A second hypothesis is that there is a trade-off between primary and secondary metabolic functions under varying levels of nitrogen, often called growth–defence trade-off (He *et al.*, 2022). At higher levels of nitrogen, plants may allocate nitrogen resources towards primary growth and reproduction (e.g. producing more flowers) and away from secondary metabolites, whereas at lower concentrations nitrogen may be allocated more towards secondary metabolites. While this trade-off has largely only been explored in the context of chemical defence to herbivores, floral scent is composed of secondary metabolites and may respond similarly, either through compositional changes (e.g. in terpenes or green-leaf volatiles) or by changes in total emissions. Alternatively, floral scent may not be resource-limited. Instead, shifts in nitrogen availability could influence floral scent through shifts in enzyme activity or gene expression that regulate volatile synthesis (Dudareva *et al.*, 2013). This study aims to clarify whether nitrogen availability in the soil influences the production of total emissions or emissions of specific compounds, with a particular focus on whether nitrogen-containing compounds, such as indole, are responsive to soil nitrogen addition.

We conducted parallel greenhouse and field experiments to examine how nitrogen addition affects floral morphology, nectar production and sugar concentration, flower production, inflorescence height and floral scent emissions in two *Ipomopsis* species and their naturally occurring hybrids in the Colorado Rocky Mountains. These *Ipomopsis* taxa have served as a model for studies of floral traits (Campbell, 2004), including floral volatile emissions (Irwin and Dorsett, 2002; Bischoff *et al.*, 2014; Campbell *et al.*, 2019; Powers *et al.*, 2022; Wu *et al.*, 2025). Floral morphological traits in these *Ipomopsis* taxa, such as corolla length, corolla width, style length, anther position and sepal width, affect a variety of biotic interactions, including pollinator attraction, pollen transfer and defence against seed predation (Campbell *et al.*, 1997, 2022*a*, *b*; Meléndez-Ackerman *et al.*, 1997). *Ipomopsis tenuituba* ssp. *tenuituba*, *Ipomopsis aggregata* ssp. *aggregata* and their hybrids occupy a broad elevational gradient with natural variation in nitrogen availability (Campbell *et al.*, 1997), providing an opportunity to assess how increased nitrogen influences floral traits in subalpine ecosystems. Comparing two closely related, hybridizing species allows us to explore how nitrogen availability might drive different responses to changing soil nutrients under climate change. Previous studies suggest that increased soil nutrients (NPK) can modify floral morphology in *I. aggregata*, with changes in corolla width and nectar production potentially increasing pollinator attraction (Burkle and Irwin, 2009). No studies, however, have investigated the impact of nitrogen on floral scent in these species. Both species emit dozens of floral VOCs, and *I. tenuituba* and hybrids, but rarely *I. aggregata*, emit indole at night (Bischoff *et al.*, 2014), providing the opportunity to explore the effects of soil nitrogen on nitrogenous floral volatiles. Indole serves to attract hawkmoth pollinators to *I. tenuituba*, playing a role in reproductive isolation between the species (Bischoff *et al.*, 2015).

We asked the following questions. (1) Does nitrogen addition affect flower morphology, flower production, inflorescence height or nectar production and concentration? (2) Does nitrogen addition affect total floral scent emission, emission composition or rates of nitrogenous compounds, and how do these effects compare in magnitude with those on other aspects of reproduction? (3) Do responses of volatiles to nitrogen addition differ among *I. aggregata*, *I. tenuituba* and hybrids, or between greenhouse and field environments?

## MATERIALS AND METHODS

### Study system

We studied two closely related species in the *Ipomopsis aggregata* species complex (Porter *et al.*, 2010), *Ipomopsis aggregata* subsp. *aggregata* and *Ipomopsis tenuituba* subsp. *tenuituba,* and their naturally occurring hybrids. These two species of *Ipomopsis* are monocarpic perennials, with *I. aggregata* in particular being widely distributed throughout the western USA (Grant and Wilken, 1988). Plants typically live as vegetative rosettes for 2–10+ years before flowering once and dying (Campbell, 1997). At our field sites, *I. aggregata* typically grows below 2900 m elevation, while *I. tenuituba* grows at elevations above 3100 m and hybrids can be found at intermediate elevations between the two species. Flowers of both species have tubular corollas but differ in colour and morphologies. *Ipomopsis aggregata* has short, wide red flowers and is primarily hummingbird-pollinated, with rare insect visitors (Campbell *et al.*, 1991), whereas *I. tenuituba* flowers have longer, narrower tubes that range in colour from pink to white and produce less nectar. Both species can be hummingbird- or hawkmoth-pollinated (Campbell *et al.*, 1997), but hummingbirds prefer to visit *I. aggregata* while the rarer hawkmoths, when present after dusk, prefer to visit *I. tenuituba* (Campbell *et al.*, 1997). Hybrids express a mixture of these traits and are preferred by hummingbirds over *I. tenuituba* (Campbell *et al.*, 2002). *Ipomopsis* typically flower for a period of 4-5 weeks (Campbell, 1989) and produce around 80–150 flowers on average for *I. aggregata* and hybrids and around 200 flowers per plant for *I. tenuituba* (Campbell and Wendlandt, 2013). In these species of *Ipomopsis* and their hybrids, the volatile profile is largely composed of terpenoids and aliphatics, with the most dominant compound being α-pinene, a monoterpene (Bischoff *et al.*, 2014). Indole, a nitrogenous volatile organic compound that is a primary attractant for hawkmoth pollinators (Bischoff *et al.*, 2015), is emitted by *I. tenuituba* and some hybrids in small amounts at night and occasionally emitted by *I. aggregata* at night.

We ran two parallel experiments examining the effect of nitrogen availability on floral scent and other floral traits: a greenhouse experiment at the Rocky Mountain Biological Laboratory (RMBL) in Gothic, Colorado (38°57′29″N, 106°59′06″W; elevation 2900 m) and a field experiment along an elevational transect at the nearby Poverty Gulch, CO, about 10 km away (elevation 2900–3100 m). The greenhouse leveraged controlled conditions to isolate specific effects of nitrogen while a complementary field experiment captured more ecologically relevant responses in a natural environment. The two experiments were originally designed separately and differed in inclusion of taxa for practical reasons. Hybrids and *I. tenuituba* both emit indole, a nitrogenous floral scent compound. Hybrids, but not *I. tenuituba*, were included in the field study because their sites are more accessible for multiple field trips than the higher-elevation *I. tenuituba* populations, which entail a long hike to visit. The greenhouse experiment instead utilized *I. tenuituba* because hybrid populations are relatively small, and destructive sampling by potting could not be justified at a continuing, long-term study site.

Nitrogen deposition in the vicinity of RMBL remains relatively low (mean = 0.4 NO_3_^−^ and 0.06 NH_4_^+^ g m^−2^ year^−1^; NADP, 2006) compared with the US average (∼1–3 g m^−2^ year^−1^ total oxidized and reduced N; EPA, 2024), which makes RMBL a useful site for assessing experimentally imposed nitrogen treatments against a low-deposition background. Many high-elevation systems are nutrient-poor and can be nitrogen-limited depending on moisture; relevant work documenting this comes from Niwot Ridge, CO (Bowman *et al.*, 1993; Theodose and Bowman, 1997).

### Experimental design

For the greenhouse experiment, we used *I. tenuituba* and *I. aggregata* to test how nitrogen affects volatile emissions. For the field experiment, we measured hybrids of *I. tenuituba × I. aggregata* and a nearby *I. aggregata* population. We collected 40 plants of *I. aggregata* and *I. tenuituba* for the greenhouse experiment and marked 77 plants at natural *I. aggregata* and hybrid populations (46 and 31 plants, respectively) in Poverty Gulch, Gunnison County, CO, for the field experiment.

#### Greenhouse experiment

On 24 June 2022 we collected bolting plants of *I. aggregata* and *I. tenuituba* from four subalpine sites with mostly parental types at the ends of a hybrid zone in Poverty Gulch, Gunnison County, CO, USA, that are assigned letters from A to L in descending order of elevation (Campbell *et al.*, 1997). We collected 20 *I. tenuituba* plants from sites A, B and C (ten, four and six individuals, respectively; elevation 3100 m) and 20 *I. aggregata* plants across US Forest Service Road 552 from site L (map in Fig. 1B in Aldridge and Campbell, 2009; elevation 2911 m). All plants were extracted with taproot and soil intact, potted immediately in 2-L pots with soil from the collection site, and thoroughly watered. Plants were transported to the RMBL and placed in an outdoor WeatherPort screenhouse (WeatherPort, Delta, CO, USA), henceforth referred to as the greenhouse experiment. Plants were watered as needed for the first week to minimize transplant shock. To control for environmental variation within the greenhouse, plants were haphazardly reassigned to new locations on the bench every week.

Six days after collection, ten plants from each species were randomly assigned to the nitrogen + water treatment and received 0.5 g of ammonium nitrate dissolved in 200 mL of water once a week for 5 weeks. The remaining plants were assigned to the water-only treatment and received an equivalent amount of water but no ammonium nitrate. Plants in both treatments were watered every other day with 200 mL of water.

#### Field experiment

In Poverty Gulch, 20 bolting plants, at least 1 m apart from each other, were tagged at both the L site (38°57′21′′N 107°04′32′′W; elevation 2911 m) and the I site (38°57′41′′N 107°05′10′′W; elevation 3020 m; Aldridge and Campbell, 2009). Half received 2 g of ammonium nitrate dissolved in 400 mL water once a week for 5 weeks. Field plants received higher concentrations of ammonium nitrate compared with greenhouse plants due to runoff, nutrient leaching into the surrounding soil, and competition for nutrient uptake by neighbours. The water-only group received an equivalent amount of water but no additional nutrients. Regardless of treatment, the water was poured slowly at the base of each plant to reduce surface runoff. We also took measurements from plants that received no additional nutrients or water, referred to as the no addition group. At the *I. aggregata* L site, additions began on 24 June 2022 and concluded the week of 19 July 2022. At the hybrid I site, nitrogen was added weekly between 28 June and 26 July 2022.

### Leaf reflectance

To evaluate uptake of nitrogen by the plants, we used reflectance spectrophotometry to estimate leaf chlorophyll content, which is typically proportional to leaf nitrogen content (Evans, 1989). Thirty-two days after the start of nitrogen additions, we collected the first two leaves below the first flower on the inflorescence from greenhouse plants. For leaves collected in the field, which were collected 5 weeks after the start of nitrogen additions, we stored the leaves on ice until reflectance was measured. We measured the leaf spectra with an Ocean Optics Red Tide USB650 reflectance spectrometer with an LS-1 light source. The chlorophyll index was calculated using the Chl NDI method in Richardson *et al.* (2002). We used this index as a relative measure of chlorophyll content for comparisons among treatments and taxa. Measurements were taken from the bottom of the leaf because variation in trichome density can interfere with accurate measurements (Levizou *et al.*, 2005).

### Floral traits

We measured ten distinct traits, including corolla length, corolla width, maximum and minimum stamen length, style length, sepal width, flower number, nectar volume and sugar concentration, and inflorescence height. These characteristics have been shown to influence plant fitness in *Ipomopsis* through a variety of mechanisms (Campbell, 1989; Campbell *et al.*, 2022*a*, *b*), such as pollen delivery, floral visitation and seed predation. Phenotypic and genetic correlations between most of these floral traits and volatiles have been previously reported (Campbell, 1996; Campbell *et al.*, 2022*a*, *b*). Whereas some traits are correlated, that does not affect conclusions as we are not examining effects of the traits on a response variable. We selected flowers for measurement if they had progressed into the female floral phase and had developed a receptive stigma. Flower morphology was measured using callipers (Campbell, 1989). Corolla length was measured as the distance from the base of the calyx to the opening of the corolla tube. Corolla width was determined from the inside diameter of the corolla tube at the opening. Style length was the distance from the base of the calyx to the stigma. Maximum and minimum stamen length were determined by measuring the distances from the highest and lowest anther to the base of the calyx. Finally, sepal width was measured as the distance across the base of an individual sepal.

In the field experiment, a total of 247 flowers were measured for morphology from 46 *I. aggregata* plants and 31 hybrids (sample sizes in Supplementary Data Table S1). Each trait was measured one to five times per plant. We measured floral traits 17–34 d after the start of nitrogen addition (Supplementary Data Table S2).

In the greenhouse experiment, floral morphology was measured for two to four flowers for each of 20 *I. aggregata* and 19 *I. tenuituba* plants (Supplementary Data Table S1). Trait measurements began 20–48 d after the start of nitrogen addition. Total flower number was counted 55–56 d after treatments started for greenhouse plants only. The total flower number was measured as all the retained calyces on the inflorescences of plants. Inflorescence height was measured in greenhouse plants using a metre stick starting at the surface of the soil. Two *I. tenuituba* plants (one from each treatment) senesced before we were able to measure morphology and inflorescence height (Supplementary Data Table S1). Field measurements of total flower number and inflorescence could not be obtained due to end-of-season workloads and logistical constraints that prevented some measurements prior to senescence.

In both experiments we measured 48-h nectar production and sugar concentration (methods in Campbell *et al.*, 1991; Campbell and Powers, 2015). For the field experiment, nectar collections were prepared using folded drinking straws to cover budding flowers for 48 h to prevent any loss of nectar to pollinator or nectar robber visits. For the greenhouse experiment, two to six flowers per plant were measured by marking elongated buds with a twist tie, since no pollinators could access the flowers in the screenhouse. In both the greenhouse and the field experiment, after 48 h the nectar amount was quantified using 32-mm long 5-μL microcapillary tubes to extract nectar from the flower. Nectar production is reported as microlitres per 24 h. Nectar sugar concentration was measured in sucrose equivalents using a refractometer.

### Scent collection

Floral scent samples were collected and analysed following the methods of Wu *et al.* (2025). Briefly, scent samples were collected by placing a nylon oven bag over a single flower and sealing the bag against the peduncle with a twist tie. After allowing headspace volatiles to equilibrate for 30 min, we made a small incision in the oven bag and inserted one end of the scent trap. The scent trap was made from a capillary tube sealed with silanized quartz wool and filled with 5 mg of Tenax TA (60–80 mesh) as the trapping agent. The other end of the scent trap was connected to a micro air sampler, which pulls air through the scent trap for 15 min with a flow rate of 100 mL min^−1^. Due to limited flowering duration in the greenhouse, floral volatile measurements were taken between 20 and 40 d after treatments began (Supplementary Data Table S2).

For greenhouse plants, we collected floral volatile samples from one to five flowers per plant during the day (0900–1500 h) and night (2000–2200 h) from 38 of the 40 plants (two *I. tenuituba* plants senesced before the start of sampling). We collected a total of 108 floral scent samples for *I. aggregata* (48 day, 60 night), 110 for *I. tenuituba* (47 day, 63 night) and 15 ambient air controls (5 day, 10 night; Supplementary Data Table S3). At the time of sampling, plants were moved out of the greenhouse to an outdoor bench to avoid cross-contamination of volatiles.

In the field experiment, we collected floral volatile samples from one to seven flowers per plant during the day (0900–1300 h) and night (2000–0000 h), with a total of 37 *I. aggregata* plants and 20 hybrid plants. At the time of sampling, three *I. aggregata* plants in the nitrogen treatment did not have open flowers and were therefore not sampled. We collected a total of 77 floral scent samples for *I. aggregata* (39 day, 38 night), 116 for hybrids (31 day, 85 night) and 19 ambient air controls (8 day, 11 night; Supplementary Data Table S3).

### VOC analysis

To test the impact of nitrogen addition on floral scent, we analysed the emissions of volatile organic compounds in *I. aggregata*, *I. tenuituba* and their hybrids during the day and night. After collection, scent samples were analysed using thermal desorption gas chromatography–mass spectrometry (GC–MS) at the RMBL following Wu *et al.* (2025). Volatiles were initially identified using Shimadzu GC–MS Postrun software to find the top match in the NIST14 library (National Institute of Science and Technology), with a minimum similarity of 80 % and confirmed by comparing retention times and mass spectra with authentic standards, when available. We used the bouquet package in R 4.2.2 to compare peak areas in floral samples with those in ambient air controls to eliminate contaminants (Eisen et al., 2022). We filtered each taxon separately to maintain taxonomic differences in volatile composition. To filter the samples, we found compounds present in floral samples that were emitted at levels at least four times the mean peak area in the ambient controls and found in at least 20 % of samples. We selected compounds with retention times between 2 and 17 min.

From the initial filtering, we identified 18 floral scent compounds from the greenhouse experiment and 20 compounds from the field experiment. Of these, 11 floral VOCs overlapped between the experiments. During this initial filtering we identified four unknown monoterpenes and labelled them monoterpenes A–D; however, monoterpenes A and D were later filtered out. We then ran quantitative integrations on 28 compounds, including indole (Bischoff *et al.*, 2014), and then removed 6 compounds from the VOC list that were known column or nylon bag residue contaminants. The final compound list after quantitative integrations included 22 volatile organic compounds (Supplementary Data Tables S5–S7). These quantitative integrations were highly sensitive and picked up very low levels of compounds, allowing the detection of indole emitted by some *I. aggregata* plants.

Floral volatiles were quantified as nanograms per hour per flower using a dilution series of seven standards (α-pinene, linalool, methyl salicylate, caryophyllene, α-farnesene, indole and hexenol) accounting for the major chemical classes of volatile organic compounds emitted by plants.

### Statistical analysis

Across all traits, we tested for effects of taxon, nitrogen treatment and their interaction and, when relevant, time of day.

#### Leaf reflectance

The effects of nitrogen on chlorophyll a concentration were analysed separately for greenhouse and field experiments using linear mixed-effects and linear models, respectively, with taxon, treatment and their interaction as fixed effects. Plant identity was included as a random effect in the greenhouse model to account for repeated sampling on individual plants using the lmer function of the R package *lme4* (Bates *et al.*, 2015).

#### Morphology and nectar traits

We ran similar models for each floral morphology trait and nectar volume and concentration against treatment, taxon and their interaction. Linear mixed models were fitted for each trait using a fixed effect of taxon and treatment and a random effect of plant ID using the lmer function of the R package lme4. We compared means in the nitrogen + water and water-only treatments (greenhouse) and the no addition treatment (field) for each trait using the *glht* function of the R package multcomp (Hothorn *et al.*, 2008), contrasting treatments within taxon and time. *P*-values were adjusted for multiple comparisons using the false discovery rate (FDR) method.

For total flower number and inflorescence height in the greenhouse only, we ran separate linear models on each trait, with a combined species and treatment factor as the fixed effect. For each model, we conducted a Type III ANOVA using the *car* package (Fox *et al.*, 2012) to test for overall treatment effects. We then performed pairwise contrasts comparing treatment level within each species using generalized linear hypothesis tests (GLHT) with FDR correction for multiple testing. Estimated marginal means were calculated for each treatment level to calculate percentage changes.

#### Total emissions

To characterize the effect of nitrogen on total scent emissions, total per-flower nitrogenous VOCs and emission of the nitrogenous compound indole for *I. aggregata* and *I. tenuituba*, we ran separate models using square-root-transformed emissions against treatment, taxon, time and their interactions with a random effect of plant ID. For the field experiment, we ran the same models for *I. aggregata* and hybrids.

For indole per-flower total emissions, total per-flower nitrogenous VOCs and overall per-flower total emissions, we performed post hoc comparisons of treatment effects within each taxon and time combination. To do so, we created a combined factor of taxon, time and treatment and refitted the model. Pairwise contrasts were calculated using the *glht* function in the *multcomp* package, and *P*-values were adjusted for multiple comparisons using the FDR method. Estimated marginal means and standard errors were obtained using the *emmeans* package (Lenth, 2018).

#### Multivariate emission composition

We performed the same multivariate analyses for both the field and greenhouse experiments, and for relative and absolute amounts of VOCs. To understand how nitrogen influences the amount of VOCs produced by plants, we ran statistical tests on the effects of treatment, taxon and time. However, since these taxa are known to produce different volatile composition at night (Bischoff *et al.*, 2014), for clarity in data visualization we separated the ordinations by time. We first analysed the effect of nitrogen on floral volatile emissions separately for each taxon and time of day using a constrained ordination method, canonical analysis of principal coordinates (CAP). To lessen the effect of outliers, we square-root transformed the mean volatile emissions per plant. We used CAP with Bray–Curtis distances as implemented in the capscale function of the R package *vegan* (Oksanen *et al.*, 2022). This ordination method is suited to discovering multivariate patterns among treatments. Then we used a permutation test (anova.cca function) to assess the effects of treatment, taxon, time of day and their interaction.

For post hoc comparisons of treatment effects within each taxon and time combination, we created a combined factor of taxon, time and treatment and refitted the model. Pairwise contrasts were calculated using the *glht* function in the *multcomp* package, and *P*-values were adjusted for multiple comparisons using the FDR method. Estimated marginal means and standard errors were obtained using the *emmeans* package.

#### Effect size

To quantify the magnitude of treatment effects on visual traits, total emissions and indole, we calculated Cohen’s *d* (standardized mean difference) for pairwise contrasts between nitrogen + water and water-only in both the greenhouse and field. Cohen’s *d* was calculated using estimated marginal means (*emmeans* function) from linear models. Standard deviations for each group were calculated from the standard errors of the estimated marginal means and the number of plants used in each estimate.

To understand how species and treatment contribute to overall floral phenotype in the greenhouse, we used CAP with Bray–Curtis distances to consider all traits together (morphology, nectar, total flower number, inflorescence height, total emissions and indole emissions), rather than individually. Then we used a permutation test (*anova.cca* function) that tests taxon, treatment and their interaction.

## RESULTS

### Leaf reflectance

Based on leaf reflectance, the leaf chlorophyll index (Chl NDI) was higher in the nitrogen + water treatment compared with water-only in both the greenhouse and field experiments (greenhouse: *χ*^2^_1_ = 28.67, *P* < 0.0001; field: *F*_2,70_ = 12.29, *P* < 0.0001). In the greenhouse, there was a significant difference in the chlorophyll index between species (ANOVA*, χ*^2^_1_ = 10.05, *P* = 0.002), with a significant species by treatment interaction (ANOVA*, χ*^2^_1_ = 5.30, *P* = 0.021) driven by a stronger response to nitrogen addition by *I. aggregata* (Supplementary Data Fig. S1). In the field, there was no detectable difference in the chlorophyll index between taxa (*F*_1,70_ = 2.68, *P* = 0.106).

### Morphology

To evaluate the effects of nitrogen addition on floral morphology, pairwise contrasts were used to compare treatment levels within each species. In the greenhouse, we did not observe a difference between the nitrogen + water and water-only treatments in any of the traits measured after correcting for FDR (GLHT, all *P* > 0.07; Fig. 1). In contrast, in the field we observed a response of corolla width to nitrogen in *I. aggregata* and to water-only in hybrids. Specifically, corolla width increased by 7 % in the nitrogen + water treatment compared with the water-only treatment at the *I. aggregata* field site (GLHT, *P* = 0.024) and increased by 15 % in hybrids in the water-only treatment compared with the no addition treatment (GLHT, *P* = 0.015; Fig. 2). We did not observe any differences in the other morphology traits from the field experiment.

**Fig. 1.**
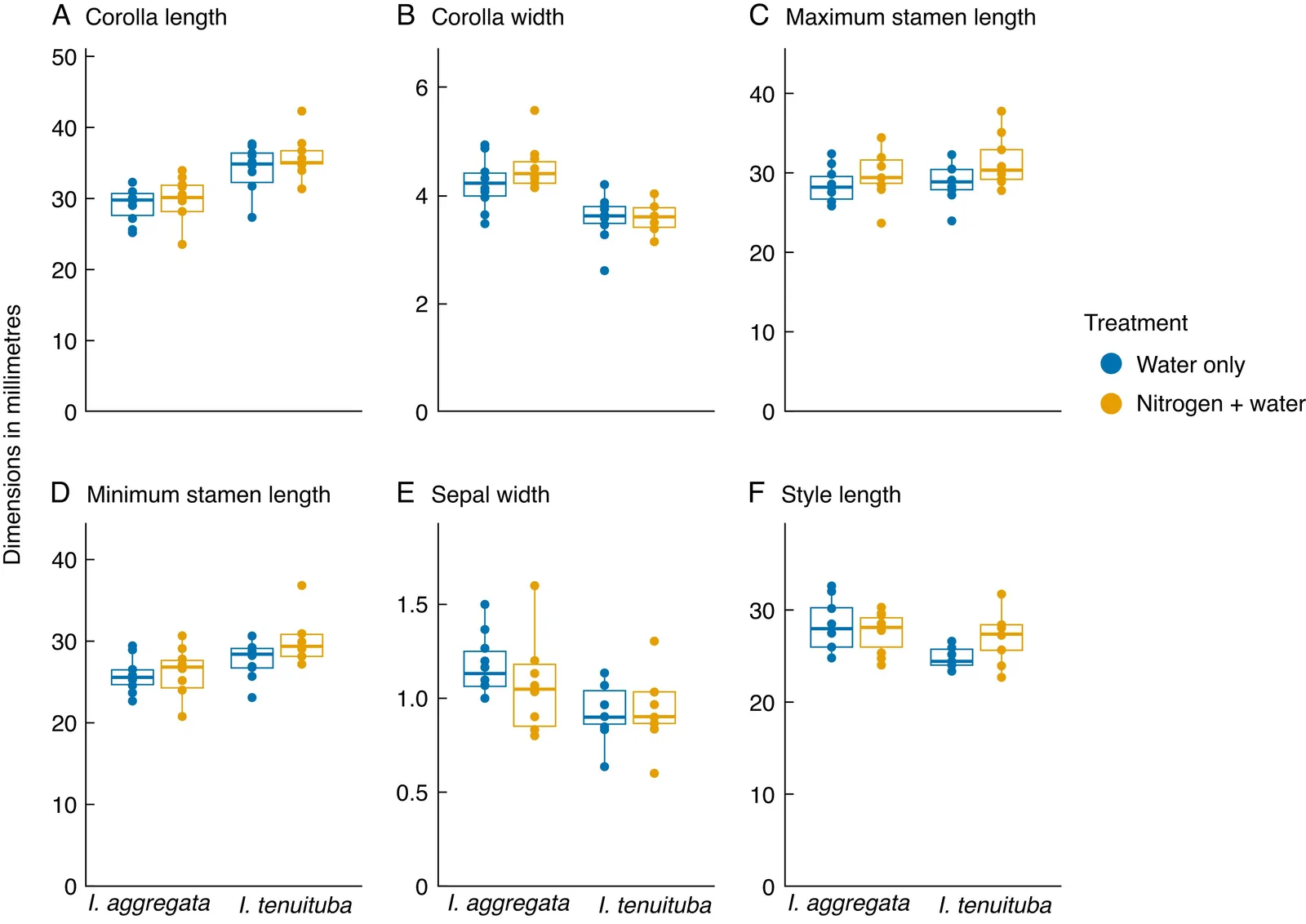
Flower morphology for *I. aggregata* and *I. tenuituba* in the greenhouse experiment. The *y*-axis shows trait length in millimetres and on the *x*-axis *I. aggregata* is on the left and *I. tenuituba* is on the right. The colour indicates treatment. Points represent plant averages. Boxes show the interquartile range with the median (horizontal line).

**Fig. 2.**
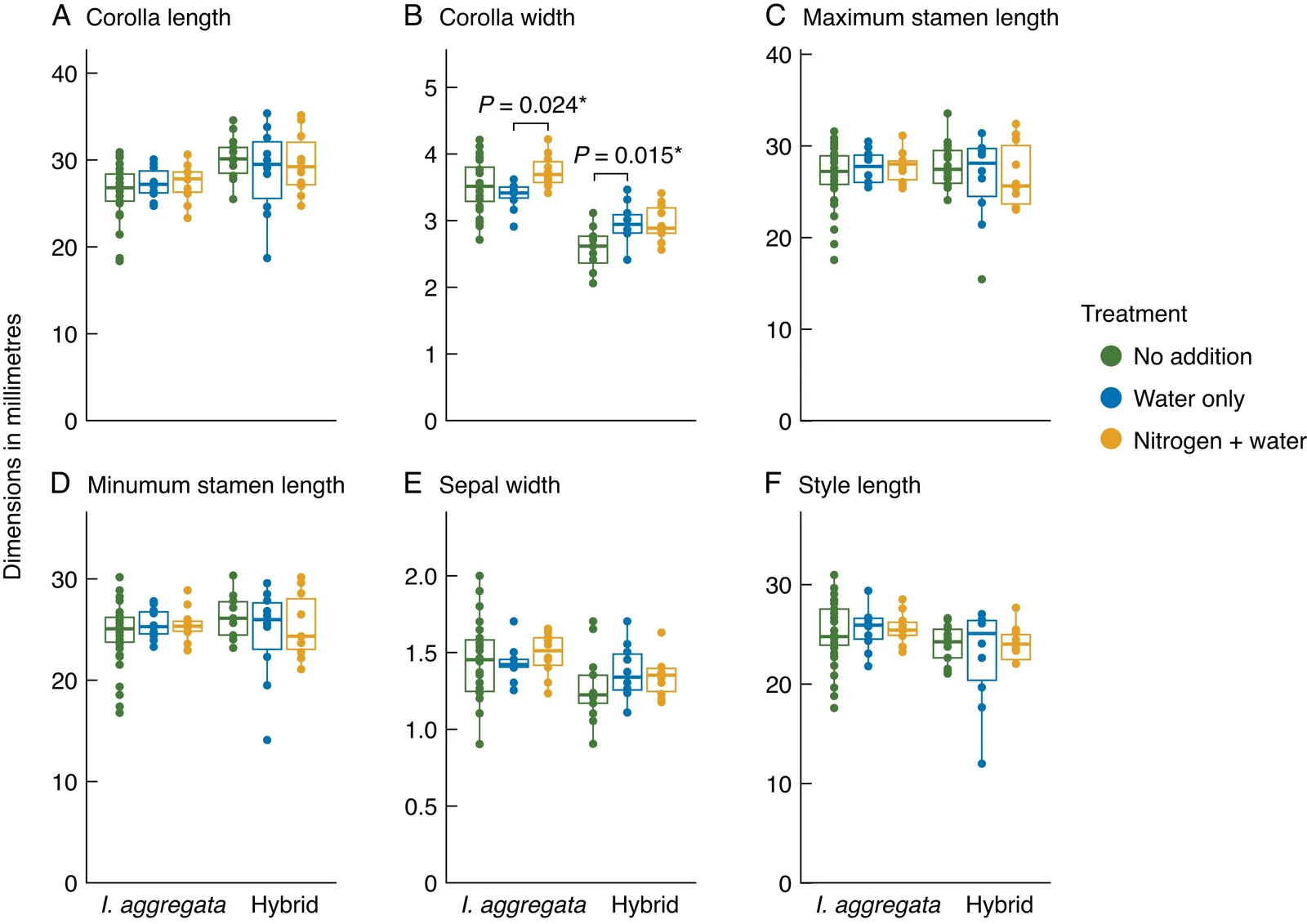
Flower morphology for *I. aggregata* and hybrids in the field experiment. Each panel represents a different floral trait. Within a single panel, the right side of the panel is hybrids and the left side is *I. aggregata*. Points represent plant averages. All traits except corolla width show no difference between the nitrogen + water, water-only and no addition treatments. Significant *P*-values from post hoc contrasts are shown above brackets. Boxes show the interquartile range with the median (horizontal line).

### Nectar

For greenhouse plants, nectar production in *I. tenuituba* decreased by 44 % under high nitrogen (ANOVA, *F*_1,18_ = 4.15, *P* = 0.042) but did not differ between treatments for *I. aggregata* (ANOVA, *F*_1,18_ = 0.838, *P* = 0.36; Fig. 3B; Supplementary Data Table S4). Nitrogen addition had no detectable effect on nectar concentration in either *I. tenuituba* (ANOVA, *F*_1,12_ = 0.003, *P* = 0.95; Fig. 3A) or *I. aggregata* (ANOVA, *F*_1,16_ = 0.813, *P* = 0.37). In the field, nitrogen addition increased nectar production in *I. aggregata* (GLHT, *P* = 0.022) by 22 % but had no detectable effect on the hybrids (ANOVA, *F*_2,25_ = 1.524, *P* = 0.467; Fig. 3D; Supplementary Data Table S4). Nectar sugar concentration did not differ across treatment groups for *I. aggregata* (ANOVA, *F*_2,39_ = 4.914, *P* = 0.31) or hybrids (ANOVA, *F*_2,55_ = 0.260, *P* = 0.87) in the field (Fig. 3C; Supplementary Data Table S4).

**Fig. 3.**
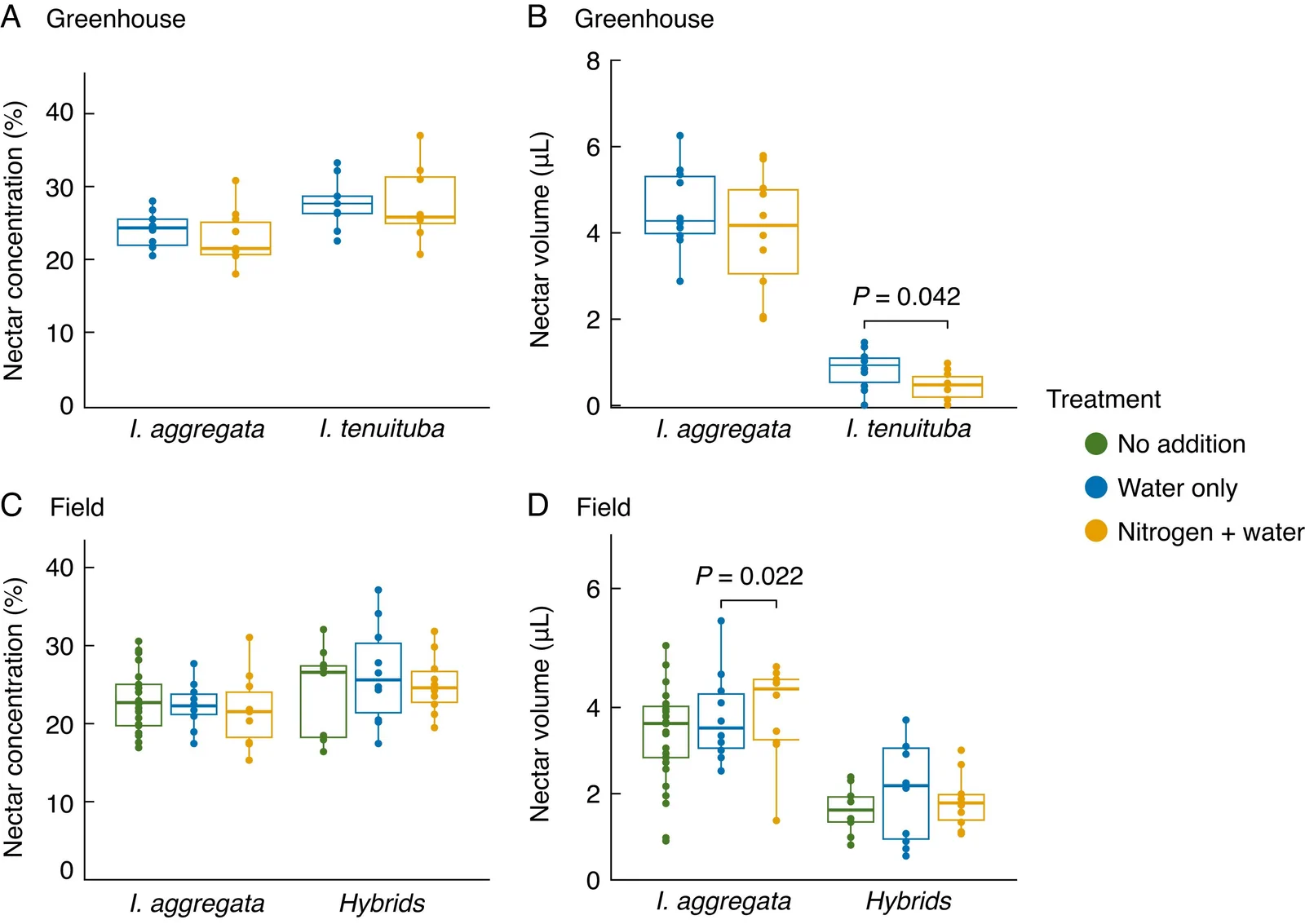
Nectar production and sugar concentration for *I. aggregata* and *I. tenuituba* in the greenhouse experiment (A, B) and for *I. aggregata* and hybrids in the field experiment (C, D). Significant *P*-values from post hoc contrasts are shown above brackets. Boxes show the interquartile range with the median (horizontal line). Points represent individual plants.

### Flower number and inflorescence height

In the greenhouse, nitrogen addition increased flower number by 71 % in *I. aggregata* (GLHT, *P* = 0.01; Fig. 4) while having a trend in the opposite direction for flower number in *I. tenuituba*, which was, however, not statistically significant (GLHT, *P* = 0.53). Similarly, inflorescence height increased by 22 % in *I. aggregata* (GLHT, *P* = 0.013) but not in *I. tenuituba* (GLHT, *P* = 0.85; Fig. 4).

**Fig. 4.**
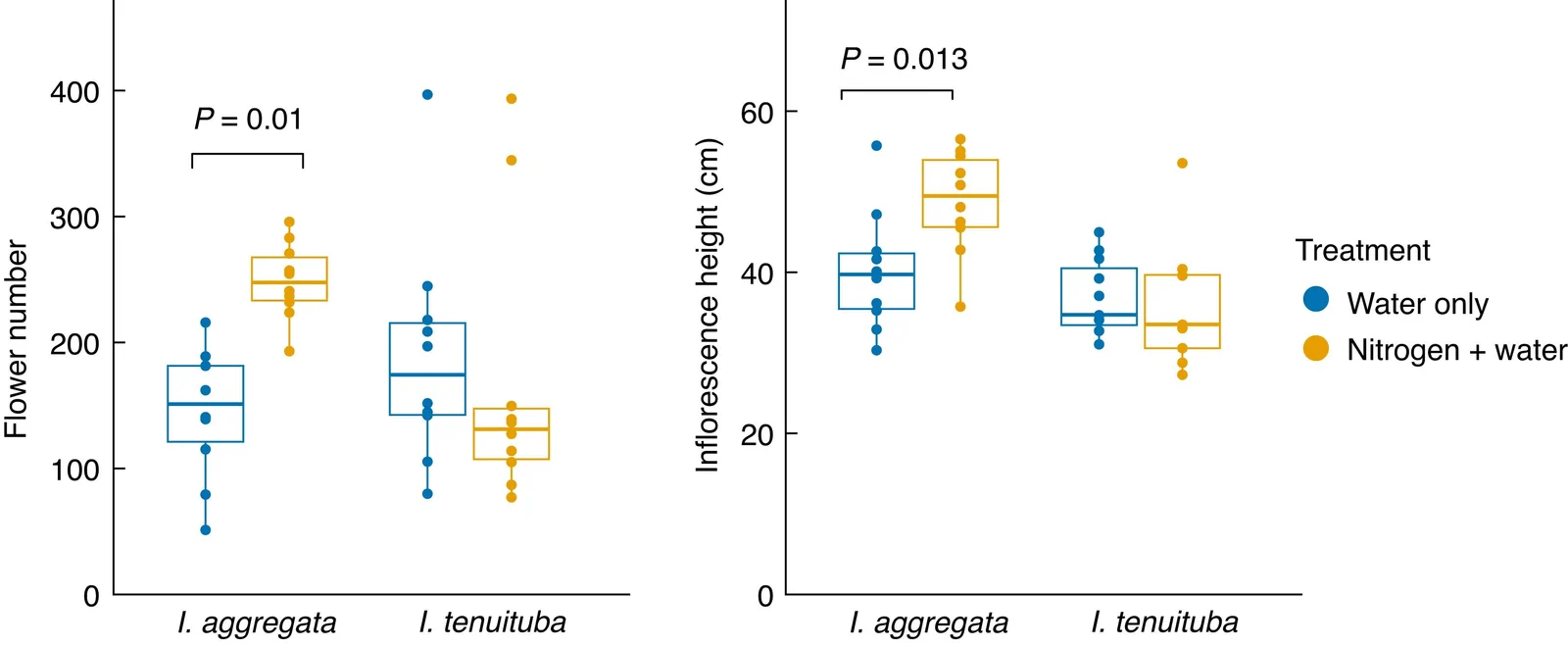
Total number of flowers produced by *I. aggregata* and *I. tenuituba* (left panel) and inflorescence height of *I. aggregata* and *I. tenuituba* in the greenhouse (right panel). Significant *P*-values for post hoc contrasts are reported over brackets. Boxes show the interquartile range with the median (horizontal line). Points represent individual plants.

### Floral scent

Across both greenhouse and field environments, floral scent in *I. aggregata*, *I. tenuituba* and hybrids was dominated by α-pinene (35 % greenhouse and 31 % field, on average) and (*E*)-β-ocimene (28 % greenhouse and 35 % field).

### Total emissions

Overall, in the greenhouse, nitrogen did not alter total per-flower volatile emissions despite variation between taxa and time of day. Time of day was the dominant predictor of total per-flower volatile emissions. Both species produced higher emissions at night (ANOVA, *χ*^2^_1_ = 24.74, *P* < 0.0001; Table 1), with nighttime emissions increasing by 292 % in *I. aggregata* and 373 % in *I. tenuituba* compared with daytime emissions (Fig. 5A). Neither nitrogen addition (ANOVA, *χ*^2^_1_ = 0.034, *P* = 0.853) nor taxonomic identity (ANOVA, *χ*^2^_1_ = 0.256, *P* = 0.613) affected total per-flower emissions, and no interaction terms were significant.

**Fig. 5.**
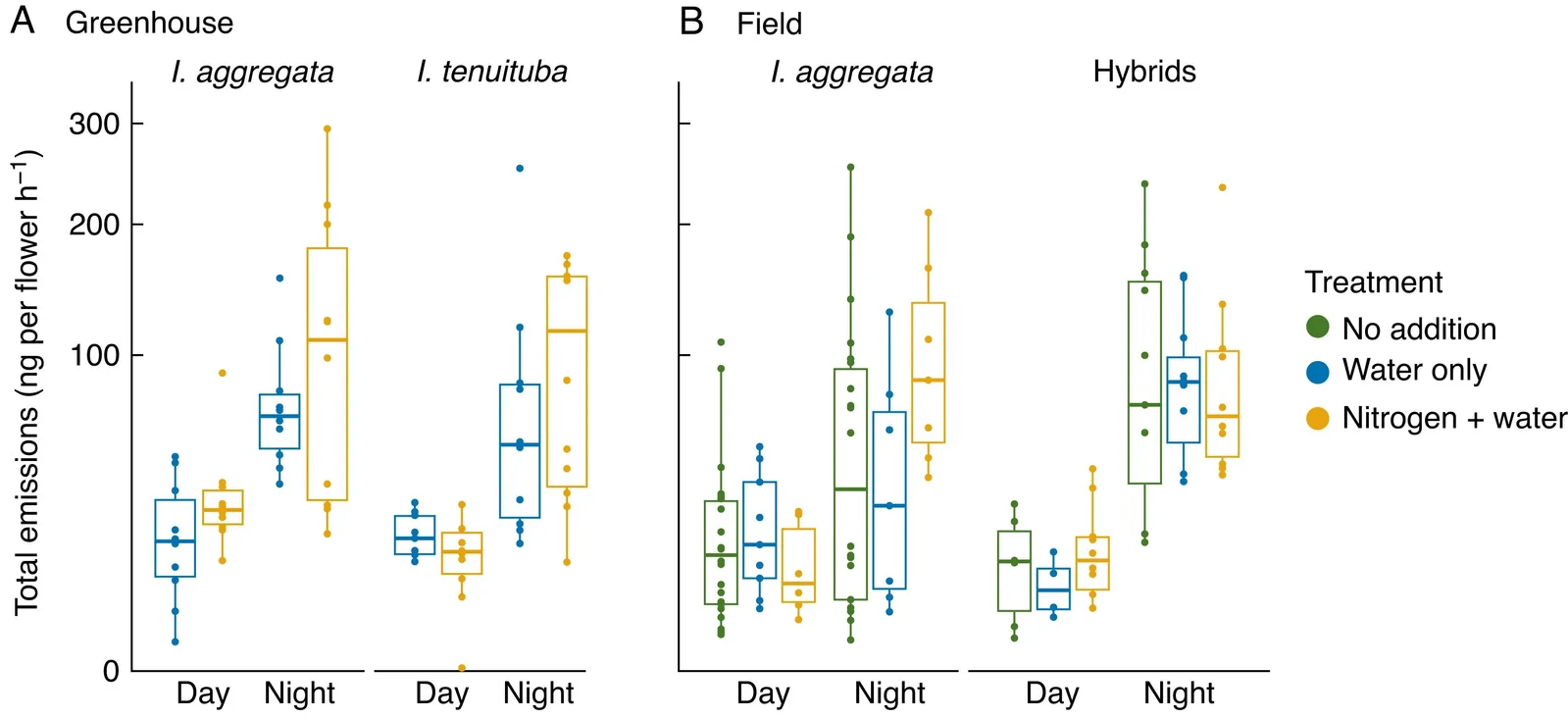
Total per-flower volatile emissions for (A) greenhouse and (B) field experiments across time (day and night), taxon and treatment levels. Points represent individual plants. (A) Greenhouse total emissions for *I. aggregata* (left panel) and *I. tenuituba* (right panel). (B) Field total emissions for *I. aggregata* (left panel) and hybrids (right panel). Time of day is on the *x*-axis; colour represents treatment. The *y*-axis is on the square-root scale. The only significant difference is between time of day for the greenhouse (*P* = 0.001) and field (*P* = 0.014, Table 2) experiments. Post hoc contrasts among treatments within species and time of day were not significant.

**Table 1.** Type III Wald *χ*^2^ tests of the effects of treatment, taxon, time and their interactions on total floral emissions in greenhouse and field experiments.

	Greenhouse			Field		
	d.f.	*χ* ^2^	*P*-value	d.f.	*χ* ^2^	*P*-value
Intercept	1	2.493	0.114	1	0.293	0.588
Treatment	1	0.034	0.853	2	0.083	0.959
Taxon	1	0.257	0.613	1	0.010	0.919
Time	1	24.742	<0.001***	1	5.987	0.014*
Treatment × taxon	1	0.090	0.765	2	0.181	0.913
Treatment × time	1	2.931	**0.087 .**	2	1.962	0.375
Taxon × time	1	0.245	0.621	1	0.074	0.786
Treatment × taxon × time	1	0.168	0.682	2	1.920	0.383

*** *P* < 0.001, **P < 0.01,*P < 0.05, .*P* < 0.1.

Although treatment by time showed the lowest *P*-value among interactions (ANOVA, *χ*^2^_1_ = 2.93, *P* = 0.087; Table 1), pairwise comparisons revealed that percentage changes associated with nitrogen addition were not statistically significant for either species during the day or night in the greenhouse (GLHT, all *P* > 0.05; Fig. 5A).

Consistent with greenhouse results, field total per-flower emissions were higher at night (ANOVA, *χ*^2^_2_ = 5.99, *P* = 0.014; Table 1), increasing by 257 % in *I. aggregata* and by 715 % in hybrids compared with daytime emissions (Fig. 5B). We detected no effects of taxon, treatment or their interactions on total emissions in the field.

Indole emission was investigated to understand treatment effects on a volatile with implications for reproductive isolation. Nitrogen addition had no detectable effect on per-flower indole emissions in either environment or in any taxon, during the day or night (Fig. 7). However, there was a trend for *I. tenuituba* to emit more indole than *I. aggregata* at night. Pairwise contrasts grouping taxa and time of day showed no difference between nitrogen treatments (GLHT, *P* > 0.6 for greenhouse and field). Total emissions of nitrogenous VOCs combined (indole and benzamide) also did not respond significantly to added nitrogen, a pattern consistent across environments and taxa (ANOVA, greenhouse and field, all *P* > 0.1; Supplementary Data Fig. S4). However, in the greenhouse, nitrogenous VOC emission increased at night (ANOVA, *χ*^2^_1_ = 17.08, *P* < 0.001). Pairwise contrasts revealed that nighttime emissions were significantly greater than daytime emissions in both *I. aggregata* (emmeans, *P* = 0.034) and *I. tenuituba* (emmeans, *P* = 0.003; Supplementary Data Fig. S4A).

### Multivariate emission composition

The greenhouse multivariate analysis of all volatile compounds showed that emission rates varied by time of day (function anova.cca on CAP, *P* = 0.001; Supplementary Data Fig. S2) and treatment (anova.cca, *P* = 0.041; Fig. 6A, B), though no species or interaction effects were detected (Table 2). The treatment effect in the combined model was driven primarily by increases in limonene, α-pinene and β-myrcene with increased nitrogen.

**Fig. 6.**
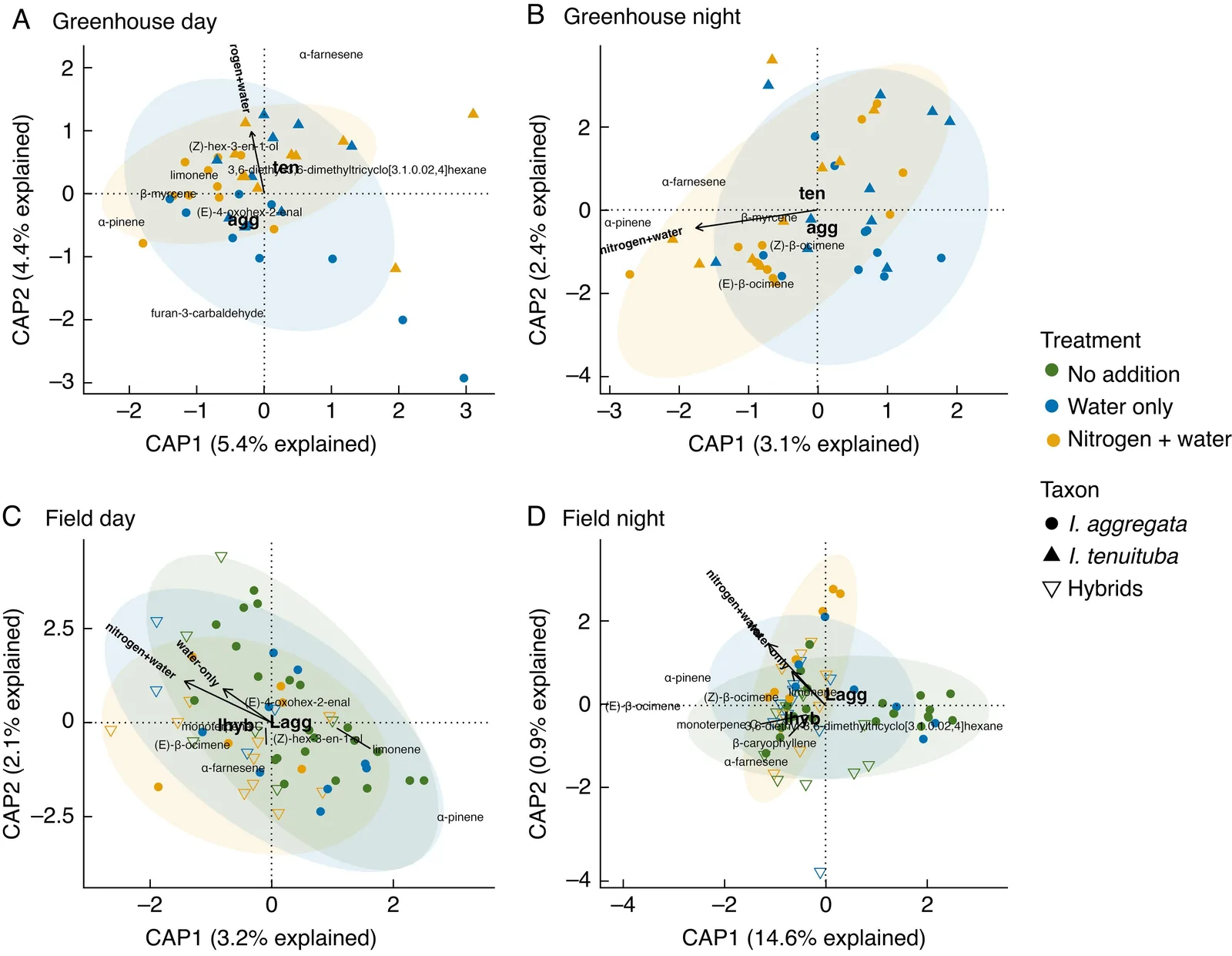
Canonical analysis of principal coordinates (CAP) ordination of volatile emission rates by time of day (day and night) in the greenhouse (top) and field (bottom) experiments. Points represent individual plants, colour indicates treatment and shapes correspond to taxa. CAP axes give percentages of variation explained. The top panels show greenhouse (A) daytime emissions and (B) nighttime emissions for *I. aggregata* and *I. tenuituba*, and the bottom panels show field (C) daytime emissions and (D) nighttime emissions for *I. aggregata* and hybrids. Taxon centroids are plotted on ordinations (agg, greenhouse *I. aggregata*; ten, greenhouse *I. tenuituba*; Lagg, field *I. aggregate*; Ihyb, field hybrids). Arrows indicate direction of treatment effects.

**Fig. 7.**
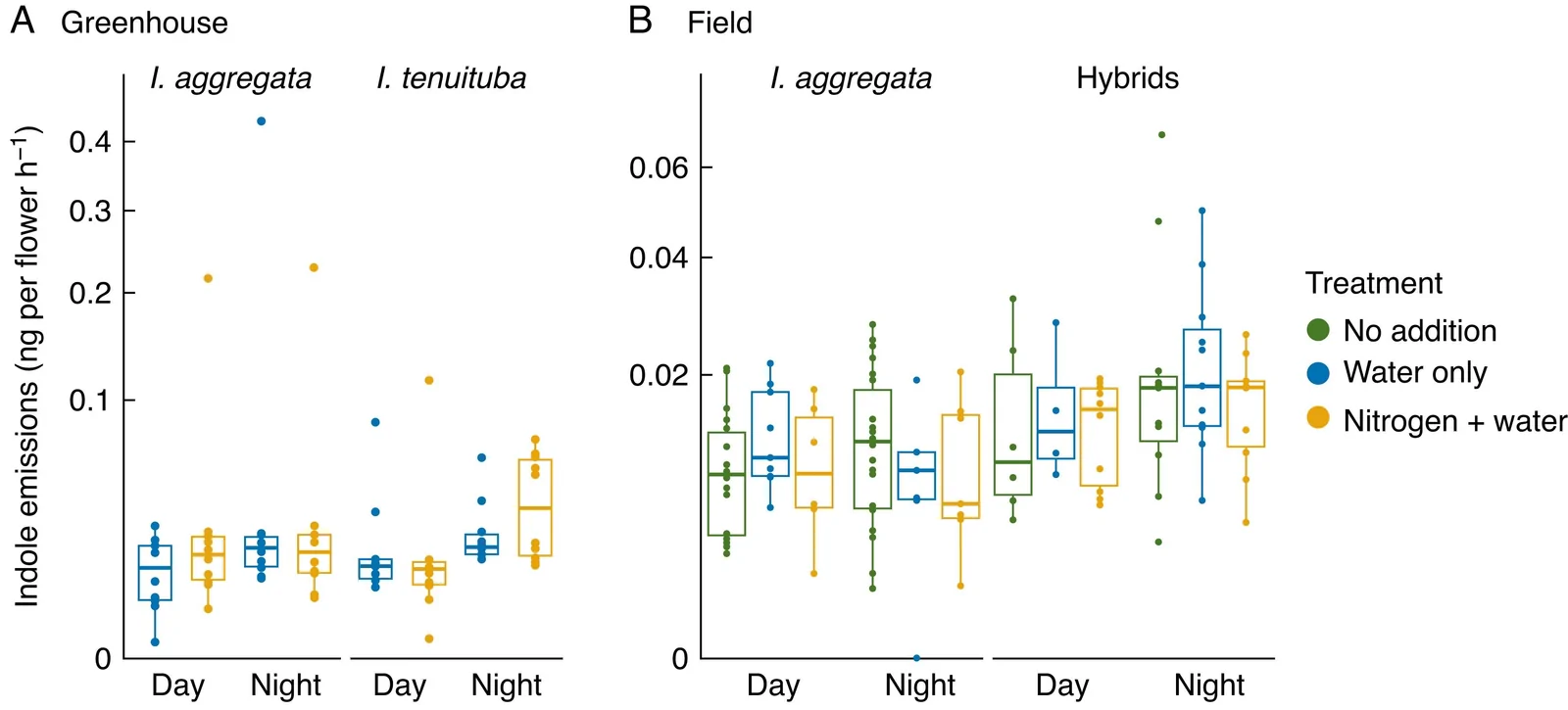
Indole emissions in nanograms per flower per hour in the (A) greenhouse and (B) field experiments by taxon, time and treatment level. (A) Greenhouse indole emissions for *I. aggregata* (left panel) and *I. tenuituba* (right panel). (B) Field indole emissions for *I. aggregata* (left panel) and hybrids (right panel). Colours indicate treatment. Taxa are shown in separate panels for each experiment. The *y*-axis has a square-root scale. Points represent individual plants.

**Table 2.** Type III ANOVA table obtained from function anova.cca on the effects of time, treatment, taxon and their interaction on a constrained ordination of principle coordinates (CAP) for emission rates in the greenhouse and field.

	Greenhouse				Field			
	d.f.	Sum of squares	*F*	*P*-value	d.f.	Sum of squares	*F*	*P*-value
Species	1	0.1352	2.6836	0.079 .	1	0.6471	12.5037	0.002**
Time	1	3.7274	73.9658	0.001***	1	3.8282	73.9732	0.001***
Treatment	1	0.1725	3.4232	0.041*	2	0.0351	2.3897	0.853
Taxa × time	1	0.0783	1.5529	0.200	1	0.1237	2.3897	0.100 .
Taxa × treatment	1	0.0325	0.6442	0.497	2	0.0917	0.8864	0.425
Time × treatment	1	0.0702	1.3924	0.227	2	0.0365	0.3531	0.812
Taxa × time × treatment	1	0.0271	0.5376	0.583	2	0.1363	1.3173	0.266
Residual	209	10.5323			181	9.3669		

***P < 0.001, **P < 0.01, *P < 0.05, .*P* < 0.1.

When day and night were analysed separately with a CAP ordination, greenhouse daytime treatment differences were associated with (*Z*)-hex-3-en-1-ol and 3,6-diethyl-3,6-dimethyltricyclo[3.1.0.0 2,4]hexane, whereas nighttime treatment differences were associated with α-pinene and β-myrcene (Fig. 6A, B).

Compound-level analyses between nitrogen + water and water-only plants in the greenhouse showed no difference in emissions in any of the compounds after correcting for FDR. Individual compound changes in both *I. aggregata* and *I. tenuituba* contribute to differences in nighttime emissions with the following compounds increasing at night: β-myrcene, (*E*)-β-ocimene, (4*E*,6*Z*)-alloocimene, monoterpene B, monoterpene C, (*Z*)-β-ocimene, α-farnesene and 3,6-diethyl-3,6-dimethyltricyclo[3.1.0.0^2,4^]hexane. Additionally, *I. aggregata* produced significantly more 3-methylbutan-1-ol compared with *I. tenuituba* in the water-only treatment (anova, *F*_1,37_ = 15.12, *P* = 0.017), but not under nitrogen + water (anova, *F*_1,38_ = 1.47, *P* = 0.79); this was the only compound to differ between species in the greenhouse.

In the field experiment, emission rates did not differ with treatment level (anova.cca, *P* = 0.853; Fig. 6C, D; Table 2). Emission rates differed by taxon when comparing hybrids with *I. aggregata* (*P* = 0.002) and by time of day (*P* = 0.001; Supplementary Data Fig. S2; Table 2). Hybrids emitted higher levels of β-myrcene, whereas field *I. aggregata* emitted more 3,6-diethyl-3,6-dimethyltricyclo[3.1.0.0^2,4^]hexane.

Field day–night differences reflected consistent compound-level shifts, with nighttime increases in (*Z*)-β-ocimene, (*E*)-β-ocimene and monoterpene C and daytime increases in (*Z*)-hex-3-en-1-ol and (E)-4-oxohex-2-enal. Compound-level analyses showed large nighttime increases across taxa in (*E*)-β-ocimene, (*Z*)-β-ocimene, α-farnesene, (4*E*,6*Z*)-alloocimene, monoterpene B, 3,6-diethyl-3,6-dimethyltricyclo[3.1.0.0^2,4^]hexane and monoterpene C, with hybrids also producing more β-myrcene and linalool at night. During the day, *I. aggregata* produced more 3-methylbutan-1-ol, similar to the result in the greenhouse experiment.

Across field taxa (ignoring time and treatment), hybrids generally emitted higher levels of indole, α-farnesene, monoterpene B, 3,6-diethyl-3,6-dimethyltricyclo[3.1.0.0^2,4^]hexane, (*Z*)-β-ocimene and (4*E*,6*Z*)-alloocimene than did *I. aggregata*.

Using relative rather than absolute emissions of VOCs, multivariate analysis in the field and greenhouse showed only a time difference (anova.cca, both greenhouse and field *P* < 0.0001) but no main effect of treatment or taxon (anova.cca, all *P* > 0.1; Supplementary Data Fig. S5).

### Effect size

Effect sizes comparing nitrogen + water and water-only plants were largest for *I. aggregata* flower number and inflorescence height in the greenhouse and *I. aggregata* corolla width in the field (Fig. 8). Notably, they were often inconsistent in direction across taxa or across environments.

**Fig. 8.**
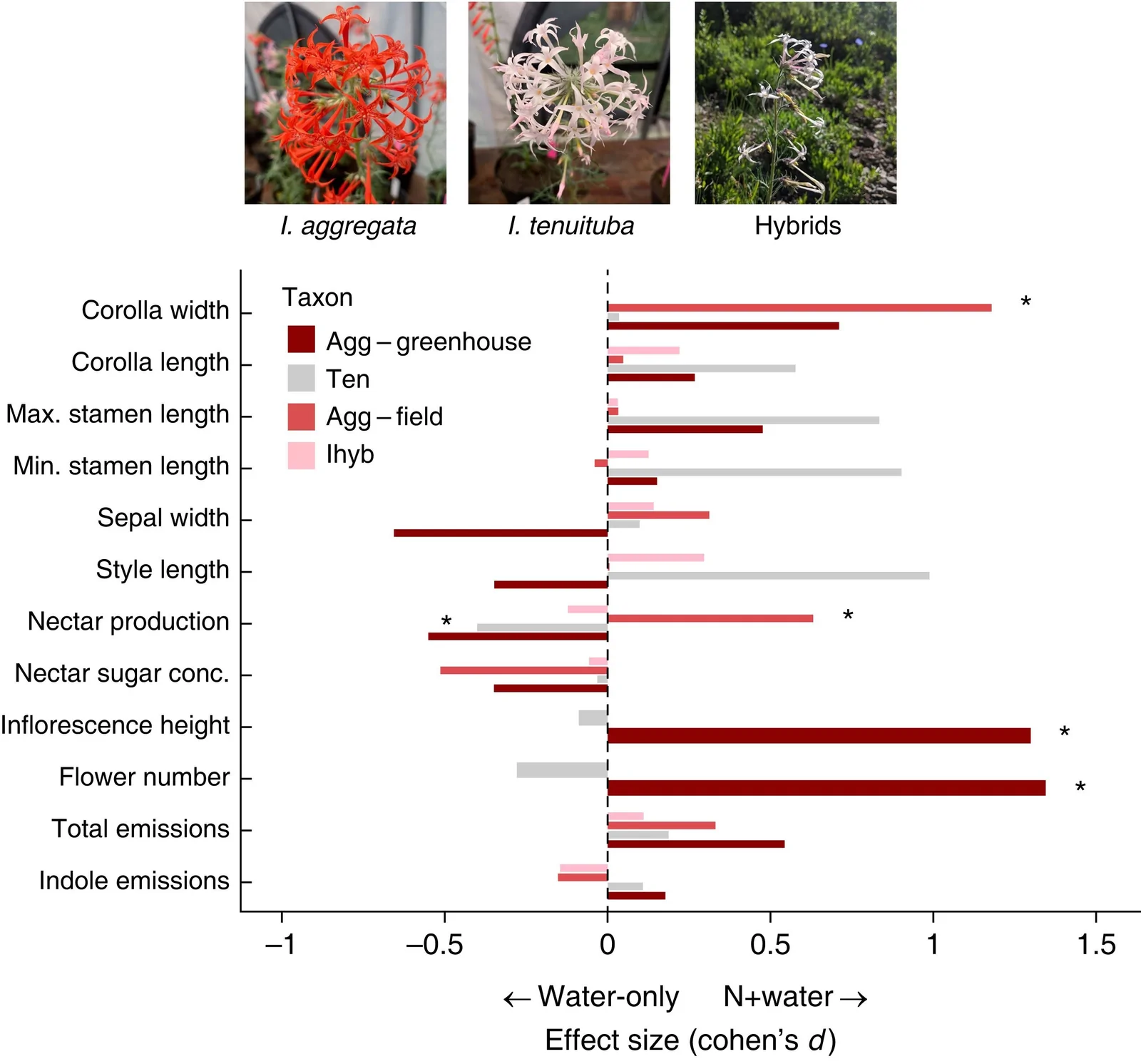
Effect size (Cohen’s *d*) for traits measured in *I. aggregata* (Agg – greenhouse) and *I. tenuituba* (Ten) in the greenhouse and *I. aggregata* (Agg – field) and hybrids (Ihyb) in the field experiment. Cohen’s *d* was calculated as the nitrogen + water treatment mean minus the water-only treatment mean, divided by the pooled standard deviation. Bars pointing to the right indicate that the nitrogen + water treatment mean was larger, while bars pointing to the left indicate that the water-only treatment mean was larger. Significant differences are indicated with an asterisk.

A constrained ordination (CAP) of all greenhouse traits showed significant multivariate differences due to species (anova.cca, *F*_1,32_ = 4.19, *P* = 0.014) with no effect of treatment (anova.cca, *F*_1,32_ = 2.34, *P* = 0.083; Supplementary Data Fig. S3). The interaction explained most of the variation (anova.cca, *F*_1,32_ = 8.88, *P* = 0.001), highlighting that treatment effects differed between species. Position along CAP1 was driven to the left by higher flower number, with smaller contributions from inflorescence height and total scent emissions. CAP2 was negatively associated with flower number and inflorescence height and positively with corolla length. This analysis reinforces that multivariate trait differences among species and nitrogen treatment are primarily driven by floral display, especially flower production, with other traits contributing less.

## DISCUSSION

Across parallel greenhouse and field experiments examining *I. aggregata*, *I. tenuituba* and their hybrids, corolla width (but no other aspects of floral morphology), nectar production and display size showed the strongest response to nitrogen addition; however, these responses differed across taxa and environments. Floral volatiles responded minimally to nitrogen addition, and only under greenhouse conditions, suggesting floral scent in these taxa may be more canalized compared with other floral traits. Leaf chlorophyll content increased with nitrogen addition in all taxa, consistent with previous work (Campbell *et al.*, 2010), confirming that plants were physiologically responsive to nitrogen addition.

Among the morphological traits we measured, only corolla width responded to our treatments, and only under field conditions. In field *I. aggregata*, nitrogen addition increased corolla width relative to water-only plants, consistent with Burkle and Irwin (2009), who found a similar response to NPK fertilization in this species. This response has direct consequences for pollination success, as flowers with wider corollas export more pollen during hummingbird visits (Campbell *et al.*, 1991) and are preferentially foraged upon by hummingbirds (Campbell *et al.*, 1997), suggesting that soil nitrogen availability can influence plant fitness in *I. aggregata* through its effects on floral morphology. In field hybrids, by contrast, corolla width responded to water availability rather than nitrogen, a pattern consistent with the drier conditions at the hybrid site (Campbell *et al.*, 2005) and well-documented water limitation in the field in several *Ipomopsis* floral traits (Campbell and Wendlandt, 2013; Waser and Price, 2016; Powers *et al.*, 2022). Notably, we detected no morphological response to nitrogen in either species in the greenhouse, where water was more available and less nitrogen was added, which aligns with evidence from other systems showing species-specific and dose-dependent corolla size responses to nitrogen even in controlled environments (e.g. *Cucumis sativus*, Vaudo *et al.*, 2022; *Petunia hybrida*, Majetic *et al.*, 2017; *Nicotiana tabacum*, Brito Vera *et al.*, 2025). That the field and greenhouse responses diverged in *I. aggregata* suggests that nitrogen effects on floral morphology may depend on broader environmental context, rather than nitrogen alone, and that controlled settings may miss ecologically meaningful morphological responses to nutrient addition.

Similar to other field studies looking at nutrient effects on *I. aggregata* (Campbell and Halama, 1993; Burkle and Irwin, 2009), we found that field *I. aggregata* plants produced more nectar when given nitrogen. Field hybrid and greenhouse *I. aggregata* nectar production did not respond to nitrogen, although nitrogen addition decreased nectar production in greenhouse *I. tenuituba*. Nectar sugar concentration did not change detectably with nitrogen in any of the taxa in either environment. These mixed results among taxa mirror a broader pattern in the literature, where responses of nectar production to soil nutrients vary widely across species (e.g. Shuel, 1957; Burkle and Irwin, 2009; reviewed in David *et al.*, 2019).

We found that nitrogen addition in the greenhouse increased floral display by making more flowers and taller inflorescences in *I. aggregata* but not in *I. tenuituba*. Similar studies on *I. aggregata* (Campbell and Halama, 1993; Burkle and Irwin, 2009) and in other flowering species (Frazee and Marquis, 1994; Havens *et al.*, 1995; Dorken and Barrett, 2004; Muñoz *et al.*, 2005; Hoover *et al.*, 2012) generally show increased flower production with nutrient addition. In a previous field study, adding nitrogen increased seed production in *I. aggregata* and hybrids but decreased seed production in *I. tenuituba* (Campbell *et al.*, 2010). Indeed, in this study we observe an overall trend that *I. tenuituba* responds differently to nitrogen compared with *I. aggregata* and hybrids.

Overall, we found that nitrogen availability plays a limited role in modulating total emissions per flower and emission rates of compounds. Nitrogen affected some greenhouse emission rates but had no effect in the field and no detectable effect on total emissions in either environment. Additionally, we observed no change in indole, a key pollinator attractant, and overall emissions of nitrogenous compounds showed no direct response to nitrogen addition. Although taxon-level differences were overall modest, differences were more pronounced in field environments between *I. aggregata* and hybrids.

In contrast, time of day strongly influences floral scent emission in *Ipomopsis*, as supported by previous literature looking at the same taxa and consistent with the main activity of hawkmoth pollinators after dusk (Bischoff *et al.*, 2014). Across both greenhouse and field conditions, nighttime emissions were higher than daytime emissions in all taxa, with plants increasing total volatile emissions by 3- to 7-fold at night. This pattern occurred across all taxa and in both environments, indicating that baseline temporal regulation exerts a much stronger influence on total per-flower emissions than nitrogen availability. For *Ipomopsis*, which is visited by both diurnal and nocturnal pollinators, these temporal shifts may function as both a defence and an attraction mechanism. Elevated scent production can enhance detectability by pollinators in low-light conditions. However, during the day, when herbivores and natural enemies are more active, amplifying chemical signalling may impose greater fitness costs than benefits (Theis *et al.*, 2007). Indeed, we found that monoterpenes and sesquiterpenes increased at night, whereas green-leaf volatiles and aliphatics were more prevalent during the day. In these cases, strong plasticity in scent production may not be favoured evolutionarily if the fitness benefits of increased emissions are low or temporally regulated.

Although the greenhouse multivariate model detected a treatment effect, this pattern did not persist when compounds were analysed individually, and was not detectable in the field. The absence of strong nitrogen effects across the two experiments suggests that floral volatile production in these taxa is either weakly resource-limited or constrained by regulatory processes that are unresponsive to short-term nutrient addition. Our findings align with prior studies that reported weak or inconsistent nutrient effects on floral scent relative to other floral traits like flower production. For example, Friberg *et al.* (2017) and Höfer *et al.* (2022) found no effect of nitrogen on per-flower emission rates. Majetic *et al.* (2017) found only a single compound (eugenol) increased with nitrogen (and genotype had a strong effect). Luizzi *et al.* (2021) reported an increase in per-flower scent with higher nutrients but also large variation tied to mating system. Similar to our findings, these studies demonstrate that taxon identity, environment or time explain most of the variation in emissions rather than nutrient addition.

Although per-flower volatile emissions were largely unaffected by nitrogen addition, nitrogen-driven increases in flower number mean that total plant-level scent output and nectar reward increased proportionally. A plant producing more flowers therefore presents a stronger olfactory signal and a greater cumulative nectar reward, even with no, or little, change in per-flower chemistry (Luizzi *et al.*, 2021). Indeed, all of the studies discussed above investigating effects of nitrogen on floral scent found an increase in flower production, and Majetic *et al.* (2017) found an increase in visitation by insect pollinators with added nitrogen. Rather than investing additional nitrogen in individual floral signals and rewards, our results suggest that *Ipomopsis* instead channels nitrogen into increasing floral production, amplifying plant-level attractiveness in a way that translates into increased pollinator visitation and, ultimately, greater reproductive success, as shown by Mitchell (1994).

Given that we did not find a direct influence of nitrogen on per-flower emissions, and only found a change in emission rates under certain environmental and taxon-specific conditions (greenhouse *I. aggregata*), it is likely that floral volatiles in *Ipomopsis* are not strictly limited by nitrogen substrate availability. Rather, what we find is that per-flower volatile production in *Ipomopsis* exhibits a higher degree of phenotypic canalization with respect to nutrients than do floral morphology, display or nectar traits. Canalization has most often been studied in the context of vegetative and floral morphological traits, where floral traits are generally considered to be more canalized than vegetative traits due to selection by pollinators (Brock and Weinig, 2007; Pélabon *et al.*, 2011). More recently the concept of canalization has been extended to floral scent (e.g. Friberg *et al.*, 2017), due to its role in plant fitness (reviewed in Raguso, 2008; Parachnowitsch, 2014). Similar to *Lithophragma bolanderi*, floral chemical phenotype in *Ipomopsis* appears less responsive to nutrients compared with other reproductive traits. This suggests that increased nitrogen availability is more likely to alter plant–pollinator interactions through changes in visual signalling and nectar rewards (more flowers, wider corollas, greater nectar) than by altering the chemical signals. Whether this buffering is maintained under more extreme or longer-duration nitrogen enrichment, or under combined stressors such as drought and high temperature, remains an important open question.

### Limitations

There are several limitations to our study that should be considered when interpreting these findings. First, our sample size was limited, which may have reduced our ability to detect subtle effects of nitrogen addition on floral traits, especially volatile emissions, which showed high variability among plants due to uncontrolled factors. Additionally, nitrogen was added after plants had already begun bolting, and floral traits could, in principle, respond to resources accumulated earlier in the life history. On average, *Ipomopsis* takes about 5 years to bloom; therefore, resource acquisition in the years leading up to flowering likely plays a role in trait response during the year of flowering. However, based on our experiment, it is evident that plants are able to respond plastically to changes in soil nutrients under very short timescales.

It is important to note that scale, duration and dosage may play a significant role in floral trait response to nitrogen. In a study looking at community-level responses to nitrogen addition over multiple years, Burkle and Irwin (2010) found that higher levels of nitrogen decreased corolla width in *I. aggregata* when nitrogen was applied at the plot level rather than to the individual plant, perhaps due to increased competition with other plant species. Many studies looking at the effects of nitrogen on floral traits are conducted over short timescales (typically less than 1 year) and under greenhouse conditions. While these controlled experiments are useful for detecting species and dose-dependent nitrogen responses (Majetic *et al.*, 2017; Vaudo *et al.*, 2022; Brito Vera *et al.*, 2025), they do not capture how plants respond in natural field settings. We observed differing responses in *I. aggregata* between the field and greenhouse conditions, highlighting the challenge of generalizing nitrogen effects from controlled experiments to natural populations. There is a notable lack of multi-year field studies evaluating how floral traits respond to sustained nutrient enrichment, which may have consequences for plant–pollinator dynamics. For example, Muñoz *et al.* (2005) reported a 3-year delay in the positive response of *Chuquiraga oppositifolia* to nitrogen. Similarly, at both the plant and plot level, Burkle and Irwin (2010) found a delayed effect of nitrogen addition on flower production. Both of these long-term nitrogen experiments found that increased flower production led to increases in pollinator visitation (Muñoz *et al.*, 2005; Burkle and Irwin, 2010), indicating that these delayed nitrogen responses indirectly affect plant–pollinator interactions. Future work in subalpine environments should look at effects of sustained low-level nitrogen deposition over several years to better understand real-world implications (Burkle and Irwin, 2010). Furthermore, increased nitrogen availability to entire communities could alter competitive dynamics, potentially favouring species that are more responsive to higher levels of nitrogen.

### Broader implications

Plants experiencing environmental change will likely face shifts in multiple abiotic factors simultaneously, rather than in isolation. Therefore, it is important to consider the combined effects of multiple factors when predicting how floral traits may respond to climate change. Currently, more studies have focused on the effects of water availability on floral traits and plant–pollinator interactions than on nitrogen. For example, a review by Kuppler and Kotowska (2021) found that drought had the largest effect on plant height, with flower diameter, corolla length and the number of flowers also showing strong responses. In contrast, the effects of nitrogen addition, reviewed by David *et al.* (2019), appear to be more species-specific, with few consistent trends across taxa or traits, except for a general increase in total nectar amino acid content in nectar. However, there is some evidence that flower production may respond non-linearly to nitrogen, with a threshold beyond which additional nitrogen no longer positively contributes to plant reproduction. For floral scent, drought and nitrogen responses are highly species-specific, making it difficult to predict future responses to climate change. Nevertheless, since nitrogen typically increases flower number, it should also increase overall scent on a per-plant basis, which could make plants more detectable from long distances.

Soil nitrogen availability can shift through multiple mechanisms, including increased atmospheric nitrogen deposition, fertilizer inputs, and climate-driven changes in microbial activity, litter decomposition and vegetation dynamics (Fowler *et al.*, 2013; Li *et al.*, 2019). Nitrogen availability varies across spatial scales (Vitousek and Howarth, 1991; Gruber and Galloway, 2008), emphasizing the need to understand how increased deposition affects plant communities in different regions. Although nitrogen deposition rates are lower in the Rocky Mountains compared with other parts of the USA (NADP, 2007), subalpine plant communities remain highly sensitive to changes in nutrient inputs due to naturally nutrient-poor soils (Bowman *et al.*, 1993; Theodose and Bowman, 1997).

This variation in soil nitrogen can have cascading effects on plant–pollinator interactions by altering both plant community composition and floral traits. At the community level, persistent nitrogen deposition has been shown to reduce plant species richness, particularly in grasslands and montane ecosystems, where it shifts communities towards fast-growing grasses that thrive in high-nitrogen conditions (David *et al.*, 2019). This shift reduces floral diversity and the availability of resources for pollinators. These compositional changes in plant species are likely to shift pollinator composition in these areas as well. For example, Stevens *et al.* (2018) found that plant species with floral structures suited to larger bees (e.g. bumblebees) and long-tongued pollinators (deep corollas), were less common in high nitrogen deposition sites. In contrast, plants visited by short-tongued pollinators were more likely to persist under higher levels of soil nitrogen. Moreover, changes in floral traits that attract specific pollinators may change who is visiting the flower or how pollinators behave on flowers. For example, hummingbirds foraging on *Ipomopsis* show preferential selection for wider corollas (Campbell *et al.*, 1997) and larger nectar rewards (Meléndez-Ackerman *et al.*, 1997). Still, there are considerable knowledge gaps on the impacts of nitrogen on higher trophic levels (Stevens *et al.*, 2018), including both pollinators and herbivores, which should be further explored through multi-year community studies looking at nutrient deposition in field environments.

## Supplementary Material

mcag192_Supplementary_Data

## Data Availability

Data and code used in this paper are available at https://github.com/jmpowers/ipomopsis-nitrogen.

## SUPPLEMENTARY DATA

Supplementary data are available at *Annals of Botany* online and consist of the following. Table S1: morphology and nectar trait sample size. Table S2: summary of scent samples taken from the start of nitrogen addition. Table S3: floral scent sample size. Table S4: nectar traits. Table S5: floral VOCs and overall relative amounts. Table S6: relative abundances of VOCs for greenhouse species. Table S7: relative VOCs for field taxa. Figure S1: leaf chlorophyll *a* concentrations. Figure S2: ordination of species and time (no treatment). Figure S3: ordination of all greenhouse traits. Figure S4: nitrogenous VOC total emissions. Figure S5: ordination of multivariate floral scent relative amounts.
