# Supplementary material for: Effects of nitrogen on floral scent and other reproductive traits in two closely related species of Ipomopsis and their hybrids
Source: Ann Bot. 2026 Jul 2;138(5):1693–709. doi: 10.1093/aob/mcag192 (PMC13715012; doi:10.1093/aob/mcag192)
Supplement: Supplementary file 1 [file mcag192_supplementary_data.docx]

## Supplementary Information

List of Supplementary tables and figures: Table 1: Morphology and nectar trait sample size. Table S2: summary of scent samples taken from the start of nitrogen addition. Table S3: floral scent sample size. Table S4: nectar traits. Table 5S: floral VOCs and overall relative amounts. Table S6: Relative VOCs for greenhouse species. Table S7: Relative VOCs for field taxa. Fig. S1: leaf chlorophyll index. Fig. S2: Ordination of species and time (no treatment). Fig. S3: Ordination of all greenhouse traits. Fig. S4: Nitrogenous VOC total emissions. Fig. S5: Ordination of multivariate floral scent relative amounts.

**Table S1:** Sample sizes (number of plants) for floral morphology, inflorescence height, nectar production, nectar concentration, and flower number measured across greenhouse (GH) and field experiments, by taxon and treatment. Taxa abbreviations: agg = greenhouse *I. aggregata*, ten = greenhouse *I. tenuituba*, Ihyb = field *Ipomopsis* hybrids, Lagg = field *I. aggregata.* Dashed lines indicate traits not measured in the field experiment.

| Exp | Taxa | Treatment | Corolla length | Corolla width | Style length | Max anther | Min anther | Sepal width | Inflo  height | Nectar production | Nectar conc. | Fower number |
| --- | --- | --- | --- | --- | --- | --- | --- | --- | --- | --- | --- | --- |
| GH | agg | nitrogen+water | 10 | 10 | 10 | 10 | 10 | 10 | 10 | 10 | 10 | 10 |
| GH | agg | water-only | 10 | 10 | 10 | 10 | 10 | 10 | 10 | 10 | 10 | 10 |
| GH | ten | nitrogen+water | 9 | 9 | 9 | 9 | 9 | 9 | 9 | 10 | 8 | 10 |
| GH | ten | water-only | 9 | 9 | 9 | 9 | 9 | 9 | 9 | 10 | 9 | 10 |
| Field | Ihyb | No addition | 11 | 10 | 10 | 11 | 10 | 10 | – | 9 | 9 | – |
| Field | Ihyb | nitrogen+water | 10 | 10 | 10 | 10 | 10 | 10 | – | 10 | 10 | – |
| Field | Ihyb | water-only | 10 | 10 | 10 | 10 | 10 | 10 | – | 10 | 10 | – |
| Field | Lagg | No addition | 27 | 26 | 27 | 27 | 27 | 27 | – | 24 | 24 | – |
| Field | Lagg | nitrogen+water | 9 | 9 | 9 | 9 | 9 | 9 | – | 9 | 9 | – |
| Field | Lagg | water-only | 9 | 9 | 9 | 9 | 9 | 9 | – | 9 | 9 | – |

**Table S2**: Number of scent samples taken, including floral and ambient air controls, since the start of the nitrogen additions (in days) for *I. aggregata, I. tenuituba* and hybrids in the field and greenhouse.

|  | Start Date | Days since start of N additions | Number of scent samples taken  floral (air) |
| --- | --- | --- | --- |
| Field | June 24th |  |  |
|  |  | 17 | 33 (3) |
|  |  | 18 | 19 (5) |
|  |  | 27 | 77 (8) |
|  |  | 33 | 52 (4) |
|  |  | 34 | 12 (0) |
| Greenhouse | June 30th |  |  |
|  |  | 20 | 47 (3) |
|  |  | 25 | 105 (6) |
|  |  | 35 | 36 (5) |
|  |  | 36 | 21 (1) |
|  |  | 42 | 7 (1) |
|  |  | 48 | 2 (1) |
|  |  |  |  |

**Table S3**: Number of scent samples collected in each treatment, taxa and time combination in the field and greenhouse environments.

| **Experiment** | **Species** | **Time** | **Total scent samples** | **Nitrogen+water** | **Water only** | **No addition** |
| --- | --- | --- | --- | --- | --- | --- |
| Field | *I. aggregata* | Day | 39 | 7 | 23 | 9 |
|  |  | Night | 38 | 7 | 24 | 7 |
|  | Hybrids | Day | 31 | 16 | 7 | 8 |
|  |  | Night | 85 | 30 | 26 | 29 |
| Greenhouse | *I. aggregata* | Day | 48 | 24 | 24 | — |
|  |  | Night | 60 | 32 | 28 | — |
|  | *I. tenuituba* | Day | 47 | 27 | 20 | — |
|  |  | Night | 63 | 31 | 32 | — |

**Table S4**: Results of ANOVA testing for effects of treatment on nectar traits for *I. aggregata*, *I. tenuituba*, and hybrids in greenhouse and field environments. Significant effects (*p* < 0.05) are shown in bold with an asterisk.

|  |  | **Nectar Production** | | |  | **Nectar Concentration** | | |
| --- | --- | --- | --- | --- | --- | --- | --- | --- |
|  |  | **DF** | **F** | **P-value** |  | **DF** | **F** | **P-value** |
| GH | *I. aggregata* | 1, 18 | 0.838 | 0.360 |  | 1, 16 | 0.813 | 0.367 |
|  | *I. tenuituba* | 1, 18 | 4.150 | **0.042 *** |  | 1, 12 | 0.003 | 0.955 |
| Field | *I. aggregata* | 2, 134 | 4.914 | 0.086 . |  | 2, 39 | 2.346 | 0.309 |
|  | Hybrids | 2, 25 | 1.524 | 0.467 |  | 2, 55 | 0.260 | 0.878 |
| Signif. codes: 0 ‘***’ 0.001 ‘**’ 0.01 ‘*’ 0.05 ‘.’ 0.1 ‘ ’ 1 | | | | | | | | |

**Table S5**: List of VOCs from quantitative integrations in greenhouse and field environments, ordered by retention time (RT), with Kovats index for each compound, average relative amount of each compound by experiment in percent and compound chemical classification.

| Compound | RT (min) | Kovats index | Field  Average Relative Amount | Greenhouse  Average Relative Amount | Chemical class |
| --- | --- | --- | --- | --- | --- |
| 3-methylbutan-1-ol | 3.815 | 739.41 | 0.13 | 0.08 | Aliphatic |
| furan-3-carbaldehyde | 5.376 | 840.16 | 3.31 | 3.45 | Aliphatic |
| (E)-hex-2-enal | 5.725 | 861.48 | 0.38 | 0.2 | Aliphatic |
| (Z)-hex-3-en-1-ol | 5.795 | 865.71 | 1.0 | 0.78 | Aliphatic |
| α-pinene | 7.205 | 948.59 | 31.07 | 35.06 | Monoterpene |
| (E)-4-oxohex-2-enal | 7.577 | 969.90 | 1.01 | 0.33 | Aldehyde |
| β-myrcene | 8.15 | 1002.50 | 3.77 | 6.46 | Monoterpene |
| limonene | 8.83 | 1041.06 | 5.42 | 8.47 | Monoterpene |
| (Z)-β-ocimene | 8.925 | 1046.46 | 4.47 | 3.38 | Monoterpene |
| (E)-β-ocimene | 9.112 | 1057.09 | 35.3 | 28.36 | Monoterpene |
| monoterpene B | 9.632 | 1086.79 | 0.24 | 0.35 | Monoterpene |
| linalool | 9.948 | 1104.99 | 0.07 | 0.16 | Monoterpene |
| (3E)-4,8-dimethylnona-1,3,7-triene | 10.214 | 1120.43 | 0.73 | 0.15 | Monoterpene |
| (4E,6Z)-alloocimene | 10.421 | 1132.53 | 0.79 | 0.95 | Monoterpene |
| monoterpene C | 10.452 | 1134.35 | 0.62 | 0.48 | Monoterpene |
| decan-2-one | 11.385 | 1190.16 | 1.68 | 1.26 | Aliphatic |
| geranial | 12.14 | 1237.21 | 0.1 | 0.36 | Monoterpene |
| indole | 12.956 | 1290.50 | 0.26 | 0.37 | Nitrogen compound |
| benzamide | 13.514 | 1328.68 | 4.54 | 1.62 | Nitrogen compound |
| β-caryophyllene | 14.777 | 1421.37 | 0.49 | 0.19 | Sesquiterpene |
| α-farnesene | 15.72 | 1497.23 | 4.12 | 7.1 | Sesquiterpene |
| 3,6-diethyl-3,6-dimethyltricyclo[3.1.0.0^2,4^]hexane | 16.797 | 1592.05 | 0.49 | 0.43 | Aliphatic |

**Table S6:** Relative abundance (%) of volatile compounds for greenhouse *I. aggregata* and *I. tenuituba* in each treatment.

| **Compound** | **Species** | ***N + water*** | ***Water-only*** |
| --- | --- | --- | --- |
| (3E)-4,8-dimethylnona-1,3,7-triene | *I. aggregata* | 0.06 | 0.36 |
|  | *I. tenuituba* | 0.15 | 0.04 |
| (4E,6Z)-alloocimene | *I. aggregata* | 0.59 | 0.54 |
|  | *I. tenuituba* | 2.02 | 0.54 |
| (E)-4-oxohex-2-enal | *I. aggregata* | 0.58 | 0.27 |
|  | *I. tenuituba* | 0.30 | 0.17 |
| (E)-β-ocimene | *I. aggregata* | 31.43 | 29.15 |
|  | *I. tenuituba* | 24.04 | 29.08 |
| (E)-hex-2-enal | *I. aggregata* | 0.38 | 0.17 |
|  | *I. tenuituba* | 0.15 | 0.09 |
| (Z)-β-ocimene | *I. aggregata* | 3.17 | 3.80 |
|  | *I. tenuituba* | 3.07 | 3.55 |
| (Z)-hex-3-en-1-ol | *I. aggregata* | 0.63 | 0.89 |
|  | *I. tenuituba* | 0.99 | 0.61 |
| 3,6-diethyl-3,6-dimethyltricyclo[3.1.0.0²,⁴]hexane | *I. aggregata* | 0.24 | 0.17 |
|  | *I. tenuituba* | 0.49 | 0.83 |
| 3-methylbutan-1-ol | *I. aggregata* | 0.06 | 0.21 |
|  | *I. tenuituba* | 0.07 | 0.01 |
| α-farnesene | *I. aggregata* | 5.00 | 3.26 |
|  | *I. tenuituba* | 9.34 | 10.64 |
| α-pinene | *I. aggregata* | 37.23 | 34.40 |
|  | *I. tenuituba* | 36.31 | 31.98 |
| β-caryophyllene | *I. aggregata* | 0.12 | 0.15 |
|  | *I. tenuituba* | 0.25 | 0.22 |
| β-myrcene | *I. aggregata* | 7.06 | 6.85 |
|  | *I. tenuituba* | 6.17 | 5.75 |
| Benzamide | *I. aggregata* | 1.21 | 1.77 |
|  | *I. tenuituba* | 1.97 | 1.55 |
| Decan-2-one | *I. aggregata* | 0.63 | 1.72 |
|  | *I. tenuituba* | 1.51 | 1.23 |
| Furan-3-carbaldehyde | *I. aggregata* | 2.39 | 5.62 |
|  | *I. tenuituba* | 2.91 | 3.05 |
| Geranial | *I. aggregata* | 0.10 | 0.48 |
|  | *I. tenuituba* | 0.25 | 0.63 |
| Indole | *I. aggregata* | 0.21 | 0.50 |
|  | *I. tenuituba* | 0.54 | 0.25 |
| Limonene | *I. aggregata* | 8.09 | 8.91 |
|  | *I. tenuituba* | 8.42 | 8.49 |
| Linalool | *I. aggregata* | 0.12 | 0.04 |
|  | *I. tenuituba* | 0.16 | 0.35 |
| Monoterpene B | *I. aggregata* | 0.31 | 0.31 |
|  | *I. tenuituba* | 0.39 | 0.40 |
| Monoterpene C | *I. aggregata* | 0.41 | 0.45 |
|  | *I. tenuituba* | 0.52 | 0.55 |

**Table S7:** Relative abundance (%) of volatile compounds for field *I. aggregata* and hybrids in each treatment.

| **Compound** | **Species** | ***N + water*** | ***Water-only*** | **No addition** |
| --- | --- | --- | --- | --- |
| (3E)-4,8-dimethylnona-1,3,7-triene | *I. aggregata* | 0.20 | 0.30 | 0.30 |
|  | Hybrid | 0.17 | 2.82 | 0.23 |
| (4E,6Z)-alloocimene | *I. aggregata* | 0.66 | 0.52 | 0.48 |
|  | Hybrid | 0.70 | 1.49 | 0.75 |
| (E)-4-oxohex-2-enal | *I. aggregata* | 1.61 | 0.81 | 2.18 |
|  | Hybrid | 0.66 | 0.29 | 0.45 |
| (E)-β-ocimene | *I. aggregata* | 37.13 | 24.27 | 26.41 |
|  | Hybrid | 36.63 | 42.99 | 42.08 |
| (E)-hex-2-enal | *I. aggregata* | 0.66 | 0.31 | 0.72 |
|  | Hybrid | 0.34 | 0.13 | 0.16 |
| (Z)-β-ocimene | *I. aggregata* | 4.12 | 3.55 | 3.68 |
|  | Hybrid | 4.37 | 5.91 | 4.73 |
| (Z)-hex-3-en-1-ol | *I. aggregata* | 2.86 | 1.36 | 1.51 |
|  | Hybrid | 0.59 | 0.30 | 0.66 |
| 3,6-diethyl-3,6-dimethyltricyclo[3.1.0.0²,⁴]hexane | *I. aggregata* | 0.12 | 0.12 | 0.16 |
|  | Hybrid | 0.49 | 1.28 | 0.38 |
| 3-methylbutan-1-ol | *I. aggregata* | 0.59 | 0.03 | 0.29 |
|  | Hybrid | 0.05 | 0.02 | 0.01 |
| α-farnesene | *I. aggregata* | 3.87 | 0.83 | 2.86 |
|  | Hybrid | 4.82 | 4.42 | 6.34 |
| α-pinene | *I. aggregata* | 28.35 | 45.44 | 34.28 |
|  | Hybrid | 32.54 | 23.23 | 27.45 |
| β-caryophyllene | *I. aggregata* | 0.29 | 0.56 | 0.48 |
|  | Hybrid | 0.52 | 0.31 | 0.70 |
| β-myrcene | *I. aggregata* | 4.43 | 4.42 | 4.71 |
|  | Hybrid | 3.28 | 3.52 | 2.82 |
| Benzamide | *I. aggregata* | 4.60 | 4.98 | 6.74 |
|  | Hybrid | 3.57 | 3.91 | 3.21 |
| Decan-2-one | *I. aggregata* | 1.50 | 1.95 | 2.12 |
|  | Hybrid | 1.59 | 1.05 | 1.86 |
| Furan-3-carbaldehyde | *I. aggregata* | 2.54 | 3.39 | 5.05 |
|  | Hybrid | 2.62 | 2.37 | 3.16 |
| Geranial | *I. aggregata* | 0.00 | 0.00 | 0.03 |
|  | Hybrid | 0.09 | 0.26 | 0.13 |
| Indole | *I. aggregata* | 0.16 | 0.18 | 0.27 |
|  | Hybrid | 0.26 | 0.38 | 0.21 |
| Limonene | *I. aggregata* | 5.44 | 6.10 | 7.00 |
|  | Hybrid | 5.61 | 4.54 | 3.53 |
| Linalool | *I. aggregata* | 0.10 | 0.04 | 0.07 |
|  | Hybrid | 0.05 | 0.08 | 0.09 |
| Monoterpene B | *I. aggregata* | 0.16 | 0.07 | 0.22 |
|  | Hybrid | 0.23 | 0.18 | 0.45 |
| Monoterpene C | *I. aggregata* | 0.63 | 0.78 | 0.46 |
|  | Hybrid | 0.81 | 0.52 | 0.62 |

**Figure S1:** Leaf chlorophyll index (Chl NDI) in (A) greenhouse *I. aggregata* and *I. tenuituba* and (B) field hybrids and *I. aggregata*. The leaf chlorophyll index was estimated with reflectance spectrophotometry.

**Figure S2:** Canonical analysis of principal coordinates (CAP) of multivariate floral scent emissions for each *species* and *time* of day (no treatment) in the (A) greenhouse and (B) field. Color is time of day and the shape represents taxa. Figure shows day/night differences (*p* = 0.001 in both greenhouse and field), and differences in field taxa (*p* = 0.01 field) with a taxa by time interaction (*p* = 0.01 field).

**Figure S3**: Canonical analysis of principal coordinates (CAP) of floral trait variation in greenhouse *I. aggregata* and *I. tenuituba* in response to *nitrogen+water* and *water-only* treatment. CAP1 explains 26.2% and CAP2 explains 4.6% of total variation. Points represent individual plants, colored by treatment and shaped by species. The arrow indicates the direction of the *nitrogen+water* treatment vector.

**Figure S4:** Total nitrogenous volatile organic compound (VOC) emissions (indole & benzamide) per-flower during the day and night for the A) greenhouse and B) field experiment. Each point represents the per-plant average emission. The y-axis is square-root transformed to improve visualization. Color indicates treatment.

**Figure S5:** Constrained ordination (CAP) of multivariate floral volatile relative amounts in the greenhouse (A, B) and field (C, D) experiments during the day (A, C) and night (B, D). Points represent individual plants, color indicates treatment and shapes correspond to species. CAP axes give the percent of variation explained. The top panels show greenhouse A) daytime emissions and B) nighttime emissions for *I. aggregata* and *I. tenuituba*, the bottom panels show field C) daytime emissions and D) nighttime emissions for *I. aggregata* and hybrids. Taxa centroids are plotted on ordinations (agg = greenhouse *I. aggregata*, ten = greenhouse *I. tenuituba*, Lagg = field *I. aggregata*, Ihyb = field hybrids). Arrows indicate direction of treatment effects.
